# Supplementary material for: Overlapping cell population expression profiling and regulatory inference in C. elegans
Source: BMC Genomics. 2016 Feb 29;17:159. doi: 10.1186/s12864-016-2482-z (PMC4772325; doi:10.1186/s12864-016-2482-z)
Supplement: Additional file 13: — Web supplement. (DOC 21 kb) [file 12864_2016_2482_MOESM13_ESM.zip › sortWeb/clusters/hier.300.clusters/280.html]

Cluster 280 

## Cluster 280

### Expression

| cnd-1 rep. 1 | cnd-1 rep. 2 | cnd-1 rep. 3 | pha-4 rep. 1 | pha-4 rep. 2 | pha-4 rep. 3 | ceh-27 | ceh-36 | ceh-6 | F21D5.9 | mir-57 | mls-2 | pal-1 | pros-1 | ttx-3 | unc-130 | hlh-16 | irx-1 | ceh-6 (+) hlh-16 (+) | ceh-6 (+) hlh-16 (-) | ceh-6 (-) hlh-16 (+) | cnd-1 singlets | pha-4 singlets | 0 | 60 | 120 | 150 | 180 | 240 | 330 | 390 | 420 | 480 | 540 | 570 | 600 | 630 | 660 | NAME | Functional description |
| --- | --- | --- | --- | --- | --- | --- | --- | --- | --- | --- | --- | --- | --- | --- | --- | --- | --- | --- | --- | --- | --- | --- | --- | --- | --- | --- | --- | --- | --- | --- | --- | --- | --- | --- | --- | --- | --- | --- | --- |
|  |  |  |  |  |  |  |  |  |  |  |  |  |  |  |  |  |  |  |  |  |  |  |  |  |  |  |  |  |  |  |  |  |  |  |  |  |  | *eak-7* | Enhancer of AKt-1 null |
|  |  |  |  |  |  |  |  |  |  |  |  |  |  |  |  |  |  |  |  |  |  |  |  |  |  |  |  |  |  |  |  |  |  |  |  |  |  | E02A10.5 |  |
|  |  |  |  |  |  |  |  |  |  |  |  |  |  |  |  |  |  |  |  |  |  |  |  |  |  |  |  |  |  |  |  |  |  |  |  |  |  | *gsk-3* | Glycogen Synthase Kinase family |
|  |  |  |  |  |  |  |  |  |  |  |  |  |  |  |  |  |  |  |  |  |  |  |  |  |  |  |  |  |  |  |  |  |  |  |  |  |  | T22F7.1 |  |
|  |  |  |  |  |  |  |  |  |  |  |  |  |  |  |  |  |  |  |  |  |  |  |  |  |  |  |  |  |  |  |  |  |  |  |  |  |  | *mek-2* | MAP kinase kinase or Erk Kinase |
|  |  |  |  |  |  |  |  |  |  |  |  |  |  |  |  |  |  |  |  |  |  |  |  |  |  |  |  |  |  |  |  |  |  |  |  |  |  | Y54G11A.9 |  |
|  |  |  |  |  |  |  |  |  |  |  |  |  |  |  |  |  |  |  |  |  |  |  |  |  |  |  |  |  |  |  |  |  |  |  |  |  |  | *slo-1* | SLOwpoke potassium channel family |
|  |  |  |  |  |  |  |  |  |  |  |  |  |  |  |  |  |  |  |  |  |  |  |  |  |  |  |  |  |  |  |  |  |  |  |  |  |  | H25P06.1 |  |
|  |  |  |  |  |  |  |  |  |  |  |  |  |  |  |  |  |  |  |  |  |  |  |  |  |  |  |  |  |  |  |  |  |  |  |  |  |  | *klp-20* | Kinesin-Like Protein |
|  |  |  |  |  |  |  |  |  |  |  |  |  |  |  |  |  |  |  |  |  |  |  |  |  |  |  |  |  |  |  |  |  |  |  |  |  |  | *soc-2* | Suppressor Of Clr |
|  |  |  |  |  |  |  |  |  |  |  |  |  |  |  |  |  |  |  |  |  |  |  |  |  |  |  |  |  |  |  |  |  |  |  |  |  |  | *dhhc-14* | DHHC-types zinc finger protein |
|  |  |  |  |  |  |  |  |  |  |  |  |  |  |  |  |  |  |  |  |  |  |  |  |  |  |  |  |  |  |  |  |  |  |  |  |  |  | *tbc-12* | TBC (Tre-2/Bub2/Cdc16) domain family |
|  |  |  |  |  |  |  |  |  |  |  |  |  |  |  |  |  |  |  |  |  |  |  |  |  |  |  |  |  |  |  |  |  |  |  |  |  |  | F17E5.2 |  |
|  |  |  |  |  |  |  |  |  |  |  |  |  |  |  |  |  |  |  |  |  |  |  |  |  |  |  |  |  |  |  |  |  |  |  |  |  |  | *sem-4* | SEx Muscle abnormal |
|  |  |  |  |  |  |  |  |  |  |  |  |  |  |  |  |  |  |  |  |  |  |  |  |  |  |  |  |  |  |  |  |  |  |  |  |  |  | *ppm-2* | Protein Phosphatase, Mg2+/Mn2+ dependent |
|  |  |  |  |  |  |  |  |  |  |  |  |  |  |  |  |  |  |  |  |  |  |  |  |  |  |  |  |  |  |  |  |  |  |  |  |  |  | F47D12.9 |  |
|  |  |  |  |  |  |  |  |  |  |  |  |  |  |  |  |  |  |  |  |  |  |  |  |  |  |  |  |  |  |  |  |  |  |  |  |  |  | *unc-115* | UNCoordinated |
|  |  |  |  |  |  |  |  |  |  |  |  |  |  |  |  |  |  |  |  |  |  |  |  |  |  |  |  |  |  |  |  |  |  |  |  |  |  | Y39E4B.6 |  |
|  |  |  |  |  |  |  |  |  |  |  |  |  |  |  |  |  |  |  |  |  |  |  |  |  |  |  |  |  |  |  |  |  |  |  |  |  |  | F28B3.1 |  |
|  |  |  |  |  |  |  |  |  |  |  |  |  |  |  |  |  |  |  |  |  |  |  |  |  |  |  |  |  |  |  |  |  |  |  |  |  |  | *max-2* | Motor AXon guidance |
|  |  |  |  |  |  |  |  |  |  |  |  |  |  |  |  |  |  |  |  |  |  |  |  |  |  |  |  |  |  |  |  |  |  |  |  |  |  | C48A7.15 |  |
|  |  |  |  |  |  |  |  |  |  |  |  |  |  |  |  |  |  |  |  |  |  |  |  |  |  |  |  |  |  |  |  |  |  |  |  |  |  | *gsa-1* | G protein, Subunit Alpha |
|  |  |  |  |  |  |  |  |  |  |  |  |  |  |  |  |  |  |  |  |  |  |  |  |  |  |  |  |  |  |  |  |  |  |  |  |  |  | *unc-11* | UNCoordinated |
|  |  |  |  |  |  |  |  |  |  |  |  |  |  |  |  |  |  |  |  |  |  |  |  |  |  |  |  |  |  |  |  |  |  |  |  |  |  | *nkcc-1* | Na-K-Cl Cotransporter homolog |
|  |  |  |  |  |  |  |  |  |  |  |  |  |  |  |  |  |  |  |  |  |  |  |  |  |  |  |  |  |  |  |  |  |  |  |  |  |  | *lin-59* | abnormal cell LINeage |
|  |  |  |  |  |  |  |  |  |  |  |  |  |  |  |  |  |  |  |  |  |  |  |  |  |  |  |  |  |  |  |  |  |  |  |  |  |  | *zyg-8* | ZYGote defective : embryonic lethal |
|  |  |  |  |  |  |  |  |  |  |  |  |  |  |  |  |  |  |  |  |  |  |  |  |  |  |  |  |  |  |  |  |  |  |  |  |  |  | R04E5.8 |  |
|  |  |  |  |  |  |  |  |  |  |  |  |  |  |  |  |  |  |  |  |  |  |  |  |  |  |  |  |  |  |  |  |  |  |  |  |  |  | T20F5.6 |  |
|  |  |  |  |  |  |  |  |  |  |  |  |  |  |  |  |  |  |  |  |  |  |  |  |  |  |  |  |  |  |  |  |  |  |  |  |  |  | *unc-73* | UNCoordinated |
|  |  |  |  |  |  |  |  |  |  |  |  |  |  |  |  |  |  |  |  |  |  |  |  |  |  |  |  |  |  |  |  |  |  |  |  |  |  | *goa-1* | G protein,O, Alpha subunit |
|  |  |  |  |  |  |  |  |  |  |  |  |  |  |  |  |  |  |  |  |  |  |  |  |  |  |  |  |  |  |  |  |  |  |  |  |  |  | R04A9.3 |  |
|  |  |  |  |  |  |  |  |  |  |  |  |  |  |  |  |  |  |  |  |  |  |  |  |  |  |  |  |  |  |  |  |  |  |  |  |  |  | C24A8.6 |  |
|  |  |  |  |  |  |  |  |  |  |  |  |  |  |  |  |  |  |  |  |  |  |  |  |  |  |  |  |  |  |  |  |  |  |  |  |  |  | *ttll-12* | Tubulin Tyrosine Ligase Like |
|  |  |  |  |  |  |  |  |  |  |  |  |  |  |  |  |  |  |  |  |  |  |  |  |  |  |  |  |  |  |  |  |  |  |  |  |  |  | *lron-7* | eLRR (extracellular Leucine-Rich Repeat) ONly |
|  |  |  |  |  |  |  |  |  |  |  |  |  |  |  |  |  |  |  |  |  |  |  |  |  |  |  |  |  |  |  |  |  |  |  |  |  |  | *spr-4* | Suppressor of PResenilin defect |
|  |  |  |  |  |  |  |  |  |  |  |  |  |  |  |  |  |  |  |  |  |  |  |  |  |  |  |  |  |  |  |  |  |  |  |  |  |  | *ptp-2* | Protein Tyrosine Phosphatase |
|  |  |  |  |  |  |  |  |  |  |  |  |  |  |  |  |  |  |  |  |  |  |  |  |  |  |  |  |  |  |  |  |  |  |  |  |  |  | *dnj-8* | DNaJ domain (prokaryotic heat shock protein) |
|  |  |  |  |  |  |  |  |  |  |  |  |  |  |  |  |  |  |  |  |  |  |  |  |  |  |  |  |  |  |  |  |  |  |  |  |  |  | *kap-1* | Kinesin-Associated Protein |
|  |  |  |  |  |  |  |  |  |  |  |  |  |  |  |  |  |  |  |  |  |  |  |  |  |  |  |  |  |  |  |  |  |  |  |  |  |  | R90.1 |  |
|  |  |  |  |  |  |  |  |  |  |  |  |  |  |  |  |  |  |  |  |  |  |  |  |  |  |  |  |  |  |  |  |  |  |  |  |  |  | ZK643.5 |  |
|  |  |  |  |  |  |  |  |  |  |  |  |  |  |  |  |  |  |  |  |  |  |  |  |  |  |  |  |  |  |  |  |  |  |  |  |  |  | *ehbp-1* | EH (Eps-15-homology) domain Binding Protein family |
|  |  |  |  |  |  |  |  |  |  |  |  |  |  |  |  |  |  |  |  |  |  |  |  |  |  |  |  |  |  |  |  |  |  |  |  |  |  | F46F11.8 |  |
|  |  |  |  |  |  |  |  |  |  |  |  |  |  |  |  |  |  |  |  |  |  |  |  |  |  |  |  |  |  |  |  |  |  |  |  |  |  | *kdin-1* | KiDINs220 (vertebrate scaffold protein) homolog |
|  |  |  |  |  |  |  |  |  |  |  |  |  |  |  |  |  |  |  |  |  |  |  |  |  |  |  |  |  |  |  |  |  |  |  |  |  |  | C33H5.17 |  |
|  |  |  |  |  |  |  |  |  |  |  |  |  |  |  |  |  |  |  |  |  |  |  |  |  |  |  |  |  |  |  |  |  |  |  |  |  |  | *mon-2* | yeast MON (monensin-resistant) homolog |
|  |  |  |  |  |  |  |  |  |  |  |  |  |  |  |  |  |  |  |  |  |  |  |  |  |  |  |  |  |  |  |  |  |  |  |  |  |  | F18H3.4 |  |
|  |  |  |  |  |  |  |  |  |  |  |  |  |  |  |  |  |  |  |  |  |  |  |  |  |  |  |  |  |  |  |  |  |  |  |  |  |  | F10G7.10 |  |
|  |  |  |  |  |  |  |  |  |  |  |  |  |  |  |  |  |  |  |  |  |  |  |  |  |  |  |  |  |  |  |  |  |  |  |  |  |  | *hbl-1* | HunchBack Like (fly gap gene related) |
|  |  |  |  |  |  |  |  |  |  |  |  |  |  |  |  |  |  |  |  |  |  |  |  |  |  |  |  |  |  |  |  |  |  |  |  |  |  | *mdt-26* | MeDiaTor |
|  |  |  |  |  |  |  |  |  |  |  |  |  |  |  |  |  |  |  |  |  |  |  |  |  |  |  |  |  |  |  |  |  |  |  |  |  |  | *atf-6* | ATF (cAMP-dependent transcription factor) family |
|  |  |  |  |  |  |  |  |  |  |  |  |  |  |  |  |  |  |  |  |  |  |  |  |  |  |  |  |  |  |  |  |  |  |  |  |  |  | *pps-1* | 3'-Phosphoadenosine 5'-Phosphosulfate Synthetase |
|  |  |  |  |  |  |  |  |  |  |  |  |  |  |  |  |  |  |  |  |  |  |  |  |  |  |  |  |  |  |  |  |  |  |  |  |  |  | F53C11.5 |  |
|  |  |  |  |  |  |  |  |  |  |  |  |  |  |  |  |  |  |  |  |  |  |  |  |  |  |  |  |  |  |  |  |  |  |  |  |  |  | C13F10.4 |  |
|  |  |  |  |  |  |  |  |  |  |  |  |  |  |  |  |  |  |  |  |  |  |  |  |  |  |  |  |  |  |  |  |  |  |  |  |  |  | *gck-2* | Germinal Center Kinase family |
|  |  |  |  |  |  |  |  |  |  |  |  |  |  |  |  |  |  |  |  |  |  |  |  |  |  |  |  |  |  |  |  |  |  |  |  |  |  | C56C10.11 |  |
|  |  |  |  |  |  |  |  |  |  |  |  |  |  |  |  |  |  |  |  |  |  |  |  |  |  |  |  |  |  |  |  |  |  |  |  |  |  | *rpm-1* | Regulator of Presynaptic Morphology |
|  |  |  |  |  |  |  |  |  |  |  |  |  |  |  |  |  |  |  |  |  |  |  |  |  |  |  |  |  |  |  |  |  |  |  |  |  |  | *crml-1* | CARMIL (Capping, ARp2/3, Myosin I Linker protein) homolog |
|  |  |  |  |  |  |  |  |  |  |  |  |  |  |  |  |  |  |  |  |  |  |  |  |  |  |  |  |  |  |  |  |  |  |  |  |  |  | *ogt-1* | O-linked GlcNAc Transferase |
|  |  |  |  |  |  |  |  |  |  |  |  |  |  |  |  |  |  |  |  |  |  |  |  |  |  |  |  |  |  |  |  |  |  |  |  |  |  | C26H9A.2 |  |
|  |  |  |  |  |  |  |  |  |  |  |  |  |  |  |  |  |  |  |  |  |  |  |  |  |  |  |  |  |  |  |  |  |  |  |  |  |  | F13E9.1 |  |
|  |  |  |  |  |  |  |  |  |  |  |  |  |  |  |  |  |  |  |  |  |  |  |  |  |  |  |  |  |  |  |  |  |  |  |  |  |  | K05F1.6 |  |
|  |  |  |  |  |  |  |  |  |  |  |  |  |  |  |  |  |  |  |  |  |  |  |  |  |  |  |  |  |  |  |  |  |  |  |  |  |  | *sta-1* | STAT transcription factor family |
|  |  |  |  |  |  |  |  |  |  |  |  |  |  |  |  |  |  |  |  |  |  |  |  |  |  |  |  |  |  |  |  |  |  |  |  |  |  | *glr-6* | GLutamate Receptor family (AMPA) |
|  |  |  |  |  |  |  |  |  |  |  |  |  |  |  |  |  |  |  |  |  |  |  |  |  |  |  |  |  |  |  |  |  |  |  |  |  |  | D1007.15 |  |
|  |  |  |  |  |  |  |  |  |  |  |  |  |  |  |  |  |  |  |  |  |  |  |  |  |  |  |  |  |  |  |  |  |  |  |  |  |  | C32D5.3 |  |
|  |  |  |  |  |  |  |  |  |  |  |  |  |  |  |  |  |  |  |  |  |  |  |  |  |  |  |  |  |  |  |  |  |  |  |  |  |  | Y40C5A.4 |  |
|  |  |  |  |  |  |  |  |  |  |  |  |  |  |  |  |  |  |  |  |  |  |  |  |  |  |  |  |  |  |  |  |  |  |  |  |  |  | EEED8.10 |  |
|  |  |  |  |  |  |  |  |  |  |  |  |  |  |  |  |  |  |  |  |  |  |  |  |  |  |  |  |  |  |  |  |  |  |  |  |  |  | *nhx-5* | Na/H eXchanger |
|  |  |  |  |  |  |  |  |  |  |  |  |  |  |  |  |  |  |  |  |  |  |  |  |  |  |  |  |  |  |  |  |  |  |  |  |  |  | *klc-2* | Kinesin Light Chain |
|  |  |  |  |  |  |  |  |  |  |  |  |  |  |  |  |  |  |  |  |  |  |  |  |  |  |  |  |  |  |  |  |  |  |  |  |  |  | F46F11.1 |  |
|  |  |  |  |  |  |  |  |  |  |  |  |  |  |  |  |  |  |  |  |  |  |  |  |  |  |  |  |  |  |  |  |  |  |  |  |  |  | Y57G11C.9 |  |
|  |  |  |  |  |  |  |  |  |  |  |  |  |  |  |  |  |  |  |  |  |  |  |  |  |  |  |  |  |  |  |  |  |  |  |  |  |  | *attf-5* | AT hook Transcription Factor family |
|  |  |  |  |  |  |  |  |  |  |  |  |  |  |  |  |  |  |  |  |  |  |  |  |  |  |  |  |  |  |  |  |  |  |  |  |  |  | *pld-1* | PhosphoLipase D |
|  |  |  |  |  |  |  |  |  |  |  |  |  |  |  |  |  |  |  |  |  |  |  |  |  |  |  |  |  |  |  |  |  |  |  |  |  |  | T28F3.5 |  |
|  |  |  |  |  |  |  |  |  |  |  |  |  |  |  |  |  |  |  |  |  |  |  |  |  |  |  |  |  |  |  |  |  |  |  |  |  |  | C17H11.2 |  |
|  |  |  |  |  |  |  |  |  |  |  |  |  |  |  |  |  |  |  |  |  |  |  |  |  |  |  |  |  |  |  |  |  |  |  |  |  |  | *sax-7* | Sensory AXon guidance |
|  |  |  |  |  |  |  |  |  |  |  |  |  |  |  |  |  |  |  |  |  |  |  |  |  |  |  |  |  |  |  |  |  |  |  |  |  |  | *rbc-1* | RaBConnectin related |
|  |  |  |  |  |  |  |  |  |  |  |  |  |  |  |  |  |  |  |  |  |  |  |  |  |  |  |  |  |  |  |  |  |  |  |  |  |  | *ccpp-1* | Cytosolic CarboxyPePtidase family |
|  |  |  |  |  |  |  |  |  |  |  |  |  |  |  |  |  |  |  |  |  |  |  |  |  |  |  |  |  |  |  |  |  |  |  |  |  |  | F41H10.3 |  |
|  |  |  |  |  |  |  |  |  |  |  |  |  |  |  |  |  |  |  |  |  |  |  |  |  |  |  |  |  |  |  |  |  |  |  |  |  |  | *marc-6* | MARCH (Membrane-Associated Ring finger (C3HC4)) homolog |
|  |  |  |  |  |  |  |  |  |  |  |  |  |  |  |  |  |  |  |  |  |  |  |  |  |  |  |  |  |  |  |  |  |  |  |  |  |  | *tps-1* | Trehalose 6-Phosphate Synthase |
|  |  |  |  |  |  |  |  |  |  |  |  |  |  |  |  |  |  |  |  |  |  |  |  |  |  |  |  |  |  |  |  |  |  |  |  |  |  | *unc-32* | UNCoordinated |
|  |  |  |  |  |  |  |  |  |  |  |  |  |  |  |  |  |  |  |  |  |  |  |  |  |  |  |  |  |  |  |  |  |  |  |  |  |  | *grk-1* | G-protein-coupled Receptor Kinase |
|  |  |  |  |  |  |  |  |  |  |  |  |  |  |  |  |  |  |  |  |  |  |  |  |  |  |  |  |  |  |  |  |  |  |  |  |  |  | *pxf-1* | PDZ eXchange Factor |
|  |  |  |  |  |  |  |  |  |  |  |  |  |  |  |  |  |  |  |  |  |  |  |  |  |  |  |  |  |  |  |  |  |  |  |  |  |  | *tag-304* | Temporarily Assigned Gene name |
|  |  |  |  |  |  |  |  |  |  |  |  |  |  |  |  |  |  |  |  |  |  |  |  |  |  |  |  |  |  |  |  |  |  |  |  |  |  | *mtm-6* | MTM (myotubularin) family |
|  |  |  |  |  |  |  |  |  |  |  |  |  |  |  |  |  |  |  |  |  |  |  |  |  |  |  |  |  |  |  |  |  |  |  |  |  |  | *pod-1* | Polarity and Osmotic sensitivity Defect |
|  |  |  |  |  |  |  |  |  |  |  |  |  |  |  |  |  |  |  |  |  |  |  |  |  |  |  |  |  |  |  |  |  |  |  |  |  |  | *unc-70* | UNCoordinated |
|  |  |  |  |  |  |  |  |  |  |  |  |  |  |  |  |  |  |  |  |  |  |  |  |  |  |  |  |  |  |  |  |  |  |  |  |  |  | *spc-1* | SPeCtrin |
|  |  |  |  |  |  |  |  |  |  |  |  |  |  |  |  |  |  |  |  |  |  |  |  |  |  |  |  |  |  |  |  |  |  |  |  |  |  | *oxi-1* | OXidative stress Induced |
|  |  |  |  |  |  |  |  |  |  |  |  |  |  |  |  |  |  |  |  |  |  |  |  |  |  |  |  |  |  |  |  |  |  |  |  |  |  | *ubr-1* | UBR E3 ubiquitin ligase homolog |
|  |  |  |  |  |  |  |  |  |  |  |  |  |  |  |  |  |  |  |  |  |  |  |  |  |  |  |  |  |  |  |  |  |  |  |  |  |  | M02E1.1 |  |
|  |  |  |  |  |  |  |  |  |  |  |  |  |  |  |  |  |  |  |  |  |  |  |  |  |  |  |  |  |  |  |  |  |  |  |  |  |  | B0041.5 |  |
|  |  |  |  |  |  |  |  |  |  |  |  |  |  |  |  |  |  |  |  |  |  |  |  |  |  |  |  |  |  |  |  |  |  |  |  |  |  | *sor-1* | SOp-2 Related (ectopic expression of Hox genes) |
|  |  |  |  |  |  |  |  |  |  |  |  |  |  |  |  |  |  |  |  |  |  |  |  |  |  |  |  |  |  |  |  |  |  |  |  |  |  | Y119C1B.10 |  |
|  |  |  |  |  |  |  |  |  |  |  |  |  |  |  |  |  |  |  |  |  |  |  |  |  |  |  |  |  |  |  |  |  |  |  |  |  |  | *trxr-2* | ThioRedoXin Reductase |
|  |  |  |  |  |  |  |  |  |  |  |  |  |  |  |  |  |  |  |  |  |  |  |  |  |  |  |  |  |  |  |  |  |  |  |  |  |  | *arr-1* | ARRestin family |
|  |  |  |  |  |  |  |  |  |  |  |  |  |  |  |  |  |  |  |  |  |  |  |  |  |  |  |  |  |  |  |  |  |  |  |  |  |  | Y119D3B.12 |  |
|  |  |  |  |  |  |  |  |  |  |  |  |  |  |  |  |  |  |  |  |  |  |  |  |  |  |  |  |  |  |  |  |  |  |  |  |  |  | F40E10.6 |  |
|  |  |  |  |  |  |  |  |  |  |  |  |  |  |  |  |  |  |  |  |  |  |  |  |  |  |  |  |  |  |  |  |  |  |  |  |  |  | B0286.1 |  |
|  |  |  |  |  |  |  |  |  |  |  |  |  |  |  |  |  |  |  |  |  |  |  |  |  |  |  |  |  |  |  |  |  |  |  |  |  |  | C24H10.2 |  |
|  |  |  |  |  |  |  |  |  |  |  |  |  |  |  |  |  |  |  |  |  |  |  |  |  |  |  |  |  |  |  |  |  |  |  |  |  |  | *linc-42* | Long Intervening Non-Coding RNA |
|  |  |  |  |  |  |  |  |  |  |  |  |  |  |  |  |  |  |  |  |  |  |  |  |  |  |  |  |  |  |  |  |  |  |  |  |  |  | *ceh-89* | C. Elegans Homeobox |
|  |  |  |  |  |  |  |  |  |  |  |  |  |  |  |  |  |  |  |  |  |  |  |  |  |  |  |  |  |  |  |  |  |  |  |  |  |  | *madd-4* | Muscle Arm Development Defective |
|  |  |  |  |  |  |  |  |  |  |  |  |  |  |  |  |  |  |  |  |  |  |  |  |  |  |  |  |  |  |  |  |  |  |  |  |  |  | Y37A1B.17 |  |
|  |  |  |  |  |  |  |  |  |  |  |  |  |  |  |  |  |  |  |  |  |  |  |  |  |  |  |  |  |  |  |  |  |  |  |  |  |  | *ephx-1* | EPHeXin (Eph-interacting GEF) homolog |
|  |  |  |  |  |  |  |  |  |  |  |  |  |  |  |  |  |  |  |  |  |  |  |  |  |  |  |  |  |  |  |  |  |  |  |  |  |  | *unc-16* | UNCoordinated |
|  |  |  |  |  |  |  |  |  |  |  |  |  |  |  |  |  |  |  |  |  |  |  |  |  |  |  |  |  |  |  |  |  |  |  |  |  |  | *unc-26* | UNCoordinated |
|  |  |  |  |  |  |  |  |  |  |  |  |  |  |  |  |  |  |  |  |  |  |  |  |  |  |  |  |  |  |  |  |  |  |  |  |  |  | *ptp-3* | Protein Tyrosine Phosphatase |
|  |  |  |  |  |  |  |  |  |  |  |  |  |  |  |  |  |  |  |  |  |  |  |  |  |  |  |  |  |  |  |  |  |  |  |  |  |  | *lst-6* | Lateral Signaling Target |
|  |  |  |  |  |  |  |  |  |  |  |  |  |  |  |  |  |  |  |  |  |  |  |  |  |  |  |  |  |  |  |  |  |  |  |  |  |  | *lad-2* | L1 CAM ADhesion molecule homolog |
|  |  |  |  |  |  |  |  |  |  |  |  |  |  |  |  |  |  |  |  |  |  |  |  |  |  |  |  |  |  |  |  |  |  |  |  |  |  | *acr-12* | AcetylCholine Receptor |
|  |  |  |  |  |  |  |  |  |  |  |  |  |  |  |  |  |  |  |  |  |  |  |  |  |  |  |  |  |  |  |  |  |  |  |  |  |  | *unc-53* | UNCoordinated |
|  |  |  |  |  |  |  |  |  |  |  |  |  |  |  |  |  |  |  |  |  |  |  |  |  |  |  |  |  |  |  |  |  |  |  |  |  |  | *unc-79* | UNCoordinated |
|  |  |  |  |  |  |  |  |  |  |  |  |  |  |  |  |  |  |  |  |  |  |  |  |  |  |  |  |  |  |  |  |  |  |  |  |  |  | T10B5.4 |  |
|  |  |  |  |  |  |  |  |  |  |  |  |  |  |  |  |  |  |  |  |  |  |  |  |  |  |  |  |  |  |  |  |  |  |  |  |  |  | *ehs-1* | Eps15 (endocytosis protein) Homologous Sequence |
|  |  |  |  |  |  |  |  |  |  |  |  |  |  |  |  |  |  |  |  |  |  |  |  |  |  |  |  |  |  |  |  |  |  |  |  |  |  | *unc-14* | UNCoordinated |
|  |  |  |  |  |  |  |  |  |  |  |  |  |  |  |  |  |  |  |  |  |  |  |  |  |  |  |  |  |  |  |  |  |  |  |  |  |  | *unc-76* | UNCoordinated |
|  |  |  |  |  |  |  |  |  |  |  |  |  |  |  |  |  |  |  |  |  |  |  |  |  |  |  |  |  |  |  |  |  |  |  |  |  |  | *erp-1* | Endophilin-Related Protein |
|  |  |  |  |  |  |  |  |  |  |  |  |  |  |  |  |  |  |  |  |  |  |  |  |  |  |  |  |  |  |  |  |  |  |  |  |  |  | *jnk-1* | Jun N-terminal Kinase |
|  |  |  |  |  |  |  |  |  |  |  |  |  |  |  |  |  |  |  |  |  |  |  |  |  |  |  |  |  |  |  |  |  |  |  |  |  |  | *jkk-1* | JNK Kinase |
|  |  |  |  |  |  |  |  |  |  |  |  |  |  |  |  |  |  |  |  |  |  |  |  |  |  |  |  |  |  |  |  |  |  |  |  |  |  | *ric-19* | Resistance to Inhibitors of Cholinesterase |
|  |  |  |  |  |  |  |  |  |  |  |  |  |  |  |  |  |  |  |  |  |  |  |  |  |  |  |  |  |  |  |  |  |  |  |  |  |  | Y57G11C.22 |  |
|  |  |  |  |  |  |  |  |  |  |  |  |  |  |  |  |  |  |  |  |  |  |  |  |  |  |  |  |  |  |  |  |  |  |  |  |  |  | F10E7.9 |  |
|  |  |  |  |  |  |  |  |  |  |  |  |  |  |  |  |  |  |  |  |  |  |  |  |  |  |  |  |  |  |  |  |  |  |  |  |  |  | *max-1* | Motor AXon guidance |
|  |  |  |  |  |  |  |  |  |  |  |  |  |  |  |  |  |  |  |  |  |  |  |  |  |  |  |  |  |  |  |  |  |  |  |  |  |  | *tag-232* | Temporarily Assigned Gene name |
|  |  |  |  |  |  |  |  |  |  |  |  |  |  |  |  |  |  |  |  |  |  |  |  |  |  |  |  |  |  |  |  |  |  |  |  |  |  | C16E9.2 |  |
|  |  |  |  |  |  |  |  |  |  |  |  |  |  |  |  |  |  |  |  |  |  |  |  |  |  |  |  |  |  |  |  |  |  |  |  |  |  | F54E4.3 |  |
|  |  |  |  |  |  |  |  |  |  |  |  |  |  |  |  |  |  |  |  |  |  |  |  |  |  |  |  |  |  |  |  |  |  |  |  |  |  | Y17G7B.22 |  |
|  |  |  |  |  |  |  |  |  |  |  |  |  |  |  |  |  |  |  |  |  |  |  |  |  |  |  |  |  |  |  |  |  |  |  |  |  |  | *svop-1* | SVOP (Synaptic Vesicle twO-related Protein) homolog |
|  |  |  |  |  |  |  |  |  |  |  |  |  |  |  |  |  |  |  |  |  |  |  |  |  |  |  |  |  |  |  |  |  |  |  |  |  |  | F54C9.11 |  |
|  |  |  |  |  |  |  |  |  |  |  |  |  |  |  |  |  |  |  |  |  |  |  |  |  |  |  |  |  |  |  |  |  |  |  |  |  |  | *eva-1* | Enhancer of unc-40 Ventral Axon guidance defects |
|  |  |  |  |  |  |  |  |  |  |  |  |  |  |  |  |  |  |  |  |  |  |  |  |  |  |  |  |  |  |  |  |  |  |  |  |  |  | F38B6.6 |  |
|  |  |  |  |  |  |  |  |  |  |  |  |  |  |  |  |  |  |  |  |  |  |  |  |  |  |  |  |  |  |  |  |  |  |  |  |  |  | F32D8.11 |  |
|  |  |  |  |  |  |  |  |  |  |  |  |  |  |  |  |  |  |  |  |  |  |  |  |  |  |  |  |  |  |  |  |  |  |  |  |  |  | *sax-3* | Sensory AXon guidance |
|  |  |  |  |  |  |  |  |  |  |  |  |  |  |  |  |  |  |  |  |  |  |  |  |  |  |  |  |  |  |  |  |  |  |  |  |  |  | C27H6.3 |  |
|  |  |  |  |  |  |  |  |  |  |  |  |  |  |  |  |  |  |  |  |  |  |  |  |  |  |  |  |  |  |  |  |  |  |  |  |  |  | *lim-4* | LIM domain family |
|  |  |  |  |  |  |  |  |  |  |  |  |  |  |  |  |  |  |  |  |  |  |  |  |  |  |  |  |  |  |  |  |  |  |  |  |  |  | F44E7.5 |  |
|  |  |  |  |  |  |  |  |  |  |  |  |  |  |  |  |  |  |  |  |  |  |  |  |  |  |  |  |  |  |  |  |  |  |  |  |  |  | F43D2.2 |  |
|  |  |  |  |  |  |  |  |  |  |  |  |  |  |  |  |  |  |  |  |  |  |  |  |  |  |  |  |  |  |  |  |  |  |  |  |  |  | F22D3.4 |  |
|  |  |  |  |  |  |  |  |  |  |  |  |  |  |  |  |  |  |  |  |  |  |  |  |  |  |  |  |  |  |  |  |  |  |  |  |  |  | R11B5.12 |  |
|  |  |  |  |  |  |  |  |  |  |  |  |  |  |  |  |  |  |  |  |  |  |  |  |  |  |  |  |  |  |  |  |  |  |  |  |  |  | Y73B6A.2 |  |
|  |  |  |  |  |  |  |  |  |  |  |  |  |  |  |  |  |  |  |  |  |  |  |  |  |  |  |  |  |  |  |  |  |  |  |  |  |  | C29A12.14 |  |
|  |  |  |  |  |  |  |  |  |  |  |  |  |  |  |  |  |  |  |  |  |  |  |  |  |  |  |  |  |  |  |  |  |  |  |  |  |  | *nas-10* | Nematode AStacin protease |
|  |  |  |  |  |  |  |  |  |  |  |  |  |  |  |  |  |  |  |  |  |  |  |  |  |  |  |  |  |  |  |  |  |  |  |  |  |  | ZK218.9 |  |
|  |  |  |  |  |  |  |  |  |  |  |  |  |  |  |  |  |  |  |  |  |  |  |  |  |  |  |  |  |  |  |  |  |  |  |  |  |  | F56E3.16 |  |
|  |  |  |  |  |  |  |  |  |  |  |  |  |  |  |  |  |  |  |  |  |  |  |  |  |  |  |  |  |  |  |  |  |  |  |  |  |  | C04G6.13 |  |
|  |  |  |  |  |  |  |  |  |  |  |  |  |  |  |  |  |  |  |  |  |  |  |  |  |  |  |  |  |  |  |  |  |  |  |  |  |  | *cyp-25A5* | CYtochrome P450 family |
|  |  |  |  |  |  |  |  |  |  |  |  |  |  |  |  |  |  |  |  |  |  |  |  |  |  |  |  |  |  |  |  |  |  |  |  |  |  | *srd-70* | Serpentine Receptor, class D (delta) |

### Phenotypes enriched

|  |  |  |  |
| --- | --- | --- | --- |
| **Group name** | **Number in cluster** | **Enrichment** | **FDR corrected p** |
| aldicarb response variant | 11 | 13.01 | 2.61e-06 |
| acetylcholinesterase inhibitor response variant | 11 | 11.94 | 6.12e-06 |
| drug hypersensitive | 10 | 11.96 | 2.97e-05 |
| aldicarb hypersensitive | 8 | 17.18 | 4.72e-05 |
| acetylcholinesterase inhibitor hypersensitive | 8 | 16.51 | 6.41e-05 |
| chemical response variant | 17 | 4.75 | 2.52e-04 |
| drug response variant | 14 | 5.56 | 4.86e-04 |
| neuronal outgrowth variant | 9 | 9.77 | 6.96e-04 |
| locomotion variant | 16 | 4.64 | 7.24e-04 |
| movement variant | 16 | 4.50 | 1.03e-03 |
| levamisole hypersensitive | 6 | 19.14 | 1.12e-03 |
| drug resistant | 11 | 6.73 | 1.36e-03 |
| neuron morphology variant | 8 | 10.66 | 1.45e-03 |
| neuron development variant | 10 | 7.36 | 1.87e-03 |
| cholinergic agonist hypersensitive | 6 | 17.07 | 2.08e-03 |
| axon outgrowth variant | 8 | 9.36 | 3.53e-03 |
| body posture variant | 7 | 11.16 | 4.57e-03 |
| activity level variant | 10 | 6.42 | 5.64e-03 |
| axon morphology variant | 7 | 10.68 | 5.96e-03 |
| neurite morphology variant | 7 | 10.53 | 6.40e-03 |
| locomotor coordination variant | 10 | 6.12 | 8.02e-03 |
| axon trajectory variant | 6 | 12.89 | 9.16e-03 |
| nervous system morphology variant | 11 | 5.26 | 1.09e-02 |
| gene expression variant | 15 | 3.80 | 1.30e-02 |
| pharyngeal pumping irregular | 4 | 24.77 | 2.02e-02 |
| organism physiology variant | 26 | 2.42 | 2.57e-02 |
| behavior variant | 21 | 2.76 | 2.62e-02 |
| organism behavior variant | 21 | 2.76 | 2.62e-02 |
| organism metabolism processing variant | 16 | 3.31 | 3.06e-02 |
| organism homeostasis metabolism variant | 25 | 2.43 | 3.27e-02 |
| coiler | 5 | 13.49 | 3.52e-02 |
| organism environmental stimulus response variant | 17 | 3.11 | 3.68e-02 |
| locomotion reduced | 8 | 6.43 | 3.82e-02 |
| aldicarb resistant (RNAi) | 10 | 4.92 | 4.05e-02 |
| acetylcholinesterase inhibitor resistant (RNAi) | 10 | 4.92 | 4.05e-02 |
| aldicarb resistant | 5 | 12.84 | 4.29e-02 |

### Anatomy terms enriched

|  |  |  |  |
| --- | --- | --- | --- |
| **Group name** | **Number in cluster** | **Enrichment** | **FDR corrected p** |
| somatic nervous system | 46 | 4.03 | 1.62e-12 |
| nerve ring | 32 | 5.82 | 8.57e-12 |
| nervous system | 55 | 2.72 | 6.41e-09 |
| cholinergic neuron | 19 | 7.46 | 8.14e-08 |
| motor neuron | 20 | 6.54 | 1.83e-07 |
| HSNR | 12 | 14.03 | 2.66e-07 |
| head | 53 | 2.49 | 2.91e-07 |
| HSNL | 12 | 13.88 | 2.94e-07 |
| HSN | 12 | 13.88 | 2.94e-07 |
| ganglion | 41 | 3.01 | 3.43e-07 |
| tail ganglion | 36 | 3.29 | 5.46e-07 |
| somatic neuron | 30 | 3.83 | 7.04e-07 |
| tail neuron | 35 | 3.25 | 1.36e-06 |
| tail | 40 | 2.91 | 1.48e-06 |
| neuron | 53 | 2.36 | 1.64e-06 |
| ventral ganglion | 14 | 8.05 | 7.89e-06 |
| ventral nerve cord | 17 | 5.63 | 3.11e-05 |
| serotonergic neuron | 13 | 7.56 | 5.84e-05 |
| dorsal nerve cord | 14 | 6.82 | 5.95e-05 |
| Cell | 62 | 1.90 | 1.43e-04 |
| ventral cord neuron | 22 | 3.86 | 1.64e-04 |
| head ganglion | 20 | 3.90 | 5.41e-04 |
| body ganglion | 14 | 5.62 | 5.61e-04 |
| CANR | 8 | 12.38 | 6.78e-04 |
| CANL | 8 | 12.38 | 6.78e-04 |
| CAN | 8 | 12.38 | 6.78e-04 |
| body region | 62 | 1.80 | 1.02e-03 |
| Anatomy | 62 | 1.77 | 1.59e-03 |
| hermaphrodite-specific | 36 | 2.28 | 3.63e-03 |
| interneuron | 15 | 4.39 | 3.99e-03 |
| muscular system | 35 | 2.29 | 4.65e-03 |
| ALMR | 7 | 11.70 | 4.83e-03 |
| ALML | 7 | 11.70 | 4.83e-03 |
| ALM | 7 | 11.70 | 4.83e-03 |
| head neuron | 38 | 2.15 | 6.72e-03 |
| C. elegans Cell and Anatomy Ontology | 67 | 1.64 | 6.76e-03 |
| touch receptor neuron | 9 | 7.52 | 6.83e-03 |
| nerve ring neuron | 13 | 4.73 | 8.18e-03 |
| Sex specific entity | 36 | 2.19 | 8.78e-03 |
| lumbar ganglion | 12 | 4.97 | 1.15e-02 |
| lumbar lateral left ganglion neuron | 11 | 5.29 | 1.56e-02 |
| lumbar lateral right ganglion neuron | 11 | 5.15 | 1.98e-02 |
| lumbar neuron | 11 | 5.10 | 2.11e-02 |
| body wall musculature | 26 | 2.50 | 2.35e-02 |
| lumbar lateral left ganglion | 11 | 4.99 | 2.49e-02 |
| RMDR | 5 | 15.95 | 2.53e-02 |
| RMDL | 5 | 15.95 | 2.53e-02 |
| lumbar lateral ganglion | 11 | 4.89 | 2.98e-02 |
| lumbar lateral right ganglion | 11 | 4.89 | 2.98e-02 |
| non-striated muscle | 26 | 2.45 | 3.10e-02 |
| muscle cell | 27 | 2.38 | 3.53e-02 |
| Organ system | 62 | 1.60 | 3.55e-02 |
| SMDVL | 5 | 14.62 | 3.69e-02 |
| SMDDR | 5 | 14.62 | 3.69e-02 |
| Function | 62 | 1.60 | 3.70e-02 |
| ALNR | 5 | 14.22 | 4.02e-02 |
| ALNL | 5 | 14.22 | 4.02e-02 |
| SMDVR | 5 | 14.22 | 4.02e-02 |
| SMDDL | 5 | 14.22 | 4.02e-02 |
| ventral pharyngeal ganglion (post) | 8 | 6.74 | 4.02e-02 |
| ALN | 5 | 14.22 | 4.02e-02 |
| body muscle cell | 22 | 2.65 | 4.06e-02 |
| SMD | 5 | 13.16 | 4.88e-02 |
| posterior lateral left ganglion | 7 | 7.67 | 4.88e-02 |

### GO terms enriched

|  |  |  |
| --- | --- | --- |
| **GO term** | **Number of genes** | **FDR-corrected p-value** |
| cellular component morphogenesis | 16 | 1.4e-05 |
| neuron differentiation | 13 | 4.7e-05 |
| cell projection morphogenesis | 10 | 2.2e-04 |
| signal transduction by phosphorylation | 6 | 7.2e-04 |
| stress-activated MAPK cascade | 4 | 1.2e-03 |
| cytoskeleton organization | 14 | 2.1e-03 |
| neurogenesis | 10 | 2.6e-03 |
| cell | 54 | 2.7e-03 |
| axon guidance | 6 | 3.2e-03 |
| positive regulation of signal transduction | 5 | 3.7e-03 |
| neuron projection extension | 5 | 3.7e-03 |
| actin filament-based process | 10 | 5.1e-03 |
| chemotaxis | 8 | 5.1e-03 |
| dendrite development | 5 | 5.3e-03 |
| axon | 8 | 6.8e-03 |
| cellular component organization or biogenesis | 28 | 7.2e-03 |
| developmental growth | 5 | 7.5e-03 |
| cell morphogenesis involved in differentiation | 6 | 8.1e-03 |
| locomotion | 24 | 1.3e-02 |
| regulation of cell morphogenesis involved in differentiation | 4 | 1.3e-02 |
| anatomical structure formation involved in morphogenesis | 10 | 1.4e-02 |
| regulation of cell growth | 4 | 1.8e-02 |
| regulation of neuron projection development | 4 | 1.8e-02 |
| cellular component movement | 6 | 1.9e-02 |
| synaptic vesicle localization | 4 | 2.3e-02 |
| cellular process | 42 | 2.4e-02 |
| establishment of vesicle localization | 4 | 2.8e-02 |
| single-organism organelle organization | 15 | 3.2e-02 |
| regulation of cell size | 4 | 4.0e-02 |
| response to external stimulus | 9 | 4.2e-02 |
| cytoplasm | 32 | 4.7e-02 |

### Expression clusters enriched

|  |  |  |  |
| --- | --- | --- | --- |
| **Group name** | **Number in cluster** | **Enrichment** | **FDR corrected p** |
| Genes with expression enriched in PVD and OLL neurons. Data sets were normalized by RMA and transcripts showing relative PVD enrichment (>= 1.5X) vs. the reference sample were identified by SAM analysis (False Discovery Rate, FDR < 1%). | 70 | 3.38 | 2.88e-19 |
| Genes expressed in embryonic motor neurons (identified by unc-4::GFP expressing cells). | 107 | 1.86 | 1.75e-13 |
| WT-Pico Pan-neural Enriched Genes, with genes found multiple times in a single dataset removed (without dups). | 50 | 3.28 | 8.24e-12 |
| Larval Pan-neural Enriched Genes. | 46 | 3.13 | 6.38e-10 |
| Genes predicted to be upregulated more than 2.0 fold in (AFD+AWB) datasets as compared to unsorted whole embryonic cells dataset. | 32 | 4.17 | 3.05e-09 |
| Expressed transcripts enriched in embryonic motor neurons (identified by unc-4::GFP expressing cells). | 32 | 3.48 | 2.71e-07 |
| Embryonic A-class motor neuron enriched genes. | 32 | 3.48 | 2.71e-07 |
| Embryonic Pan-neural Enriched Genes. | 41 | 2.66 | 1.64e-06 |
| Potental DAF-12 target genes identified by ChIP-chip analysis performed on strain ALF4 [daf-12 | 57 | 1.92 | 7.38e-05 |
| Genes upregulated by alg-1(-). | 34 | 2.51 | 1.51e-04 |
| miRNA targets that are significantly enriched at L1 larva stage. To generate a global view of the dynamics of miRNA-mediated regulation of gene expression during C. elegans development, authors analyzed the mRNAs in the AIN-2-GFP IP results from five developmental stages. The magnitude of the combined interaction of miRNAs with a given target mRNA was assessed by measuring the fold enrichment of that mRNA in AIN-2 IP samples, relative to the abundance of the mRNA in the corresponding total lysate. Because this enrichment in the IP sample versus total lysate directly reflects the miRISC-associated fraction of a given mRNA, high enrichment indicates the likelihood of strong miRNA-mediated regulation of the mRNA, whereas low or negative enrichment indicates the likelihood of weak or absent miRNA regulation of the mRNA. It is also possible that poor enrichment could reflect interactions that occur only in a rare subset of cells at any given stage of development. Transcripts that were significantly enriched (0 | 28 | 2.85 | 1.73e-04 |
| miRNA targets that are significantly enriched at embryo stage. To generate a global view of the dynamics of miRNA-mediated regulation of gene expression during C. elegans development, authors analyzed the mRNAs in the AIN-2-GFP IP results from five developmental stages. The magnitude of the combined interaction of miRNAs with a given target mRNA was assessed by measuring the fold enrichment of that mRNA in AIN-2 IP samples, relative to the abundance of the mRNA in the corresponding total lysate. Because this enrichment in the IP sample versus total lysate directly reflects the miRISC-associated fraction of a given mRNA, high enrichment indicates the likelihood of strong miRNA-mediated regulation of the mRNA, whereas low or negative enrichment indicates the likelihood of weak or absent miRNA regulation of the mRNA. It is also possible that poor enrichment could reflect interactions that occur only in a rare subset of cells at any given stage of development. Transcripts that were significantly enriched (0 | 23 | 3.28 | 1.90e-04 |
| Genes downregulated more than 2 fold after 24 hours of AgNPs exposure. | 27 | 2.68 | 7.91e-04 |
| Genes that are dosage compensated. | 15 | 4.22 | 9.89e-04 |
| Genes enriched in HLH-1 heat shock dataset. | 44 | 2.00 | 1.04e-03 |
| miRNA targets that are significantly enriched at L4 larva stage. To generate a global view of the dynamics of miRNA-mediated regulation of gene expression during C. elegans development, authors analyzed the mRNAs in the AIN-2-GFP IP results from five developmental stages. The magnitude of the combined interaction of miRNAs with a given target mRNA was assessed by measuring the fold enrichment of that mRNA in AIN-2 IP samples, relative to the abundance of the mRNA in the corresponding total lysate. Because this enrichment in the IP sample versus total lysate directly reflects the miRISC-associated fraction of a given mRNA, high enrichment indicates the likelihood of strong miRNA-mediated regulation of the mRNA, whereas low or negative enrichment indicates the likelihood of weak or absent miRNA regulation of the mRNA. It is also possible that poor enrichment could reflect interactions that occur only in a rare subset of cells at any given stage of development. Transcripts that were significantly enriched (0 | 22 | 3.01 | 1.23e-03 |
| Larval A-class motor neuron enriched genes. | 15 | 3.91 | 2.27e-03 |
| Genes that showed expression levels higher than the corresponding reference sample (embryonic 0hr reference). WBPaper00037950:BAG-neuron\_expressed | 69 | 1.58 | 2.46e-03 |
| Genes significantly enriched (> 2x, FDR < 5%) in a particular cell-type versus a reference sample of all cells at the same stage. WBPaper00037950:A-class-motor-neurons\_larva\_enriched | 20 | 3.06 | 2.60e-03 |
| Gene significantly up-regulated by treatment with 2.0mM of HuminFeed until older adult stage (11 days), with a minimum fold change in gene expression of 1.25. | 19 | 2.95 | 6.47e-03 |
| Genes that showed expression levels higher than the corresponding reference sample (embryonic 24hr reference). WBPaper00037950:AVA-neuron\_expressed | 67 | 1.54 | 8.08e-03 |
| Genes expressed in N2. | 108 | 1.28 | 9.77e-03 |
| Genes significantly enriched (> 2x, FDR < 5%) in a particular cell-type versus a reference sample of all cells at the same stage. WBPaper00037950:GABAergic-motor-neurons\_embryo\_enriched | 13 | 3.79 | 1.09e-02 |
| Genes depleted in muscle cells (0hr muscle dataset). Dissociated myo-3::GFP embryos were cultured for 0 hours before FACS sorting. | 33 | 2.03 | 1.33e-02 |
| FBF-associated probe sets (FDR <2.25%) | 57 | 1.59 | 1.78e-02 |
| Genes differentially expressed under EtBr treatment without UVC exposure vs after UVC exposure but without EtBr treatment at the -1h timepoint (just prior to the third UVC dose (48h)). | 50 | 1.67 | 1.81e-02 |
| mixed oogenesis/somatic | 17 | 2.83 | 2.53e-02 |
| mRNAs that were significantly enriched in the AIN-1 immunoprecipitation samples, compared to the control total mRNAs in the input extracts (p < 0.01). | 42 | 1.77 | 2.55e-02 |
| A complete list of the genes that showed differential expression in a slr-2 mutant strain. | 32 | 1.96 | 3.18e-02 |
| Developmentally modulated gene cluster. cgc4386\_cluster\_6\_5 | 10 | 4.00 | 4.57e-02 |

### Motifs enriched

|  |  |  |  |  |  |
| --- | --- | --- | --- | --- | --- |
| **Motif** | **Logo** | **Possible orthologs** | **Number of motifs in cluster** | **Enrichment** | **FDR corrected p** |
| MA0543.1 |  | eor-1 (0.55) daf-8 | 91 | 2.07 | 3.7e-12 |
| Blimp-1\_SANGER\_5\_FBgn0035625 |  | blmp-1 | 117 | 1.52 | 1.4e-08 |
| MA0535.1 |  | pax-2 daf-8 F45H11.6 | 78 | 1.97 | 2.1e-08 |
| NR2F6\_f1 |  | nhr-239 nhr-62 nhr-2 | 93 | 1.74 | 3.3e-08 |
| ZNF75A\_1 |  | ztf-3 lag-1 F26F4.8 | 81 | 1.89 | 4.9e-08 |
| HIF1A\_si |  | hif-1 (0.64) | 73 | 2.01 | 5.4e-08 |
| SP1\_f2 |  | klf-1 klf-2 ZC328.2 | 83 | 1.85 | 6.3e-08 |
| Mv109 |  | pax-2 pax-3 | 77 | 1.91 | 1.3e-07 |
| K562\_EGR1\_HudsonAlpha |  | klf-1 klf-2 ZC328.2 | 62 | 2.19 | 1.3e-07 |
| TBX1\_1 |  | tbx-38 mab-9 tbx-39 tbx-43 tbx-42 | 108 | 1.55 | 1.5e-07 |
| Atf1\_3026 |  | crh-1 | 93 | 1.70 | 1.5e-07 |
| MA0118.1 |  | ref-2 | 75 | 1.93 | 1.7e-07 |
| FOXO6\_3 |  | daf-16 (0.63) ZC328.2 | 77 | 1.90 | 1.8e-07 |
| Smad3\_3805 |  | daf-8 | 54 | 2.36 | 2.5e-07 |
| POU3F2\_2 |  | sox-4 (0.62) ceh-6 ceh-18 tbp-1 | 114 | 1.48 | 2.7e-07 |
| ALX3\_3 |  | alr-1 ceh-14 cfi-1 ZC204.2 | 22 | 5.09 | 2.9e-07 |
| MA0098.2 |  | lin-1 C24A1.2 | 105 | 1.56 | 3.0e-07 |
| MAX\_1 |  | mxl-1 K11D2.4 | 60 | 2.18 | 3.2e-07 |
| pTH10722 |  | eor-1 (0.55) ref-2 egrh-3 | 76 | 1.88 | 3.4e-07 |
| SP4\_f1 |  | Y53H1A.2 (0.58) klf-2 plp-2 | 72 | 1.94 | 3.5e-07 |
| HMGA1\_f1 |  | let-381 mel-28 mef-2 Y116A8C.22 Y61A9LA.9 | 116 | 1.46 | 3.9e-07 |
| GABPA\_f1 |  | lin-1 C24A1.2 | 102 | 1.57 | 5.3e-07 |
| pTH3220 |  | hif-1 (0.64) Y5F2A.4 (0.61) ztf-3 ceh-9 daf-12 | 54 | 2.30 | 5.4e-07 |
| V$FREAC3\_01 |  | let-381 lin-31 | 103 | 1.56 | 6.1e-07 |
| ARNT2\_si |  | aha-1 (0.59) C46E10.9 | 75 | 1.87 | 6.2e-07 |
| SMAD3\_f1 |  | daf-8 | 75 | 1.87 | 6.4e-07 |
| MA0163.1 |  | Y53H1A.2 (0.58) C09F5.3 D1081.8 | 69 | 1.96 | 6.7e-07 |
| pTH5924 |  | nhr-255 | 91 | 1.67 | 6.7e-07 |
| ZBTB7A\_1 |  | ztf-14 ref-2 lin-1 ZC328.2 | 74 | 1.87 | 9.2e-07 |
| Sp4\_1011 |  | sptf-3 klf-1 klf-2 | 87 | 1.70 | 9.8e-07 |
| MEF2B\_1 |  | mef-2 | 100 | 1.57 | 1.1e-06 |
| MA0264.1 |  | ceh-24 (0.67) dsc-1 ceh-22 | 84 | 1.73 | 1.2e-06 |
| ZBT7A\_f1 |  | klf-1 ZC328.2 | 71 | 1.90 | 1.2e-06 |
| VENTX\_1 |  | ceh-36 alr-1 ceh-53 pha-2 ceh-45 dve-1 | 78 | 1.78 | 1.8e-06 |
| HLH29 |  | aha-1 (0.59) lin-22 hlh-27 hlh-28 | 67 | 1.94 | 2.0e-06 |
| pTH9173 |  | efl-2 (0.54) | 72 | 1.86 | 2.2e-06 |
| Zfp161\_2858 |  | pzf-1 | 50 | 2.31 | 2.4e-06 |
| ONECUT2\_1 |  | ceh-48 (0.9) dsc-1 | 106 | 1.49 | 2.9e-06 |
| MA0470.1 |  | efl-1 F49E12.6 | 89 | 1.64 | 3.5e-06 |
| MA0502.1 |  | nfya-2 (0.57) ceh-20 dro-1 cey-3 lin-31 | 88 | 1.65 | 3.5e-06 |
| pTH9097 |  | Y116A8C.22 | 121 | 1.37 | 3.5e-06 |
| CXXC1\_si |  | F52B11.1 F21D5.4 | 85 | 1.67 | 4.1e-06 |
| pTH9951 |  | mex-6 pal-1 | 93 | 1.59 | 4.3e-06 |
| SP3\_1 |  | klf-1 klf-2 | 84 | 1.68 | 4.3e-06 |
| pTH9165 |  | ztf-27 | 39 | 2.67 | 4.3e-06 |
| ftz\_FlyReg\_FBgn0001077 |  | ceh-9 alr-1 lim-7 ceh-31 ceh-1 ceh-43 lin-39 ceh-16 | 79 | 1.74 | 4.4e-06 |
| fd64A\_SANGER\_5\_FBgn0004895 |  | daf-16 (0.63) fkh-10 (0.57) fkh-8 (0.52) let-381 pha-4 lin-31 fkh-7 | 84 | 1.68 | 4.6e-06 |
| pTH10042 |  | nhr-5 daf-12 | 92 | 1.59 | 5.4e-06 |
| POU3F3\_3 |  | ceh-18 unc-86 | 100 | 1.52 | 5.9e-06 |
| MA0007.2 |  | npax-1 nhr-255 lin-14 | 80 | 1.71 | 7.3e-06 |
| Sox1\_1 |  | grh-1 (-0.65) sox-4 (0.62) | 79 | 1.71 | 7.7e-06 |
| PBX1\_do |  | ceh-20 ceh-12 lin-39 | 96 | 1.55 | 8.1e-06 |
| Pax7\_3783 |  | alr-1 lim-7 ceh-18 | 76 | 1.74 | 8.7e-06 |
| K562\_ZBTB7A\_HudsonAlpha |  | ZC328.2 | 72 | 1.79 | 9.6e-06 |
| V$ZID\_01 |  | skn-1 (0.67) ztf-28 | 87 | 1.62 | 1.0e-05 |
| FOXB1\_1 |  | nhr-68 lin-31 F55C5.11 | 95 | 1.54 | 1.1e-05 |
| MA0481.1 |  | daf-16 (0.63) fkh-10 (0.57) fkh-8 (0.52) let-381 lin-31 fkh-7 | 112 | 1.41 | 1.2e-05 |
| MA0482.1 |  | elt-1 ztf-29 | 96 | 1.53 | 1.2e-05 |
| pTH5914 |  | attf-1 | 42 | 2.42 | 1.3e-05 |
| MA0451.1 |  | php-3 nhr-2 | 91 | 1.57 | 1.4e-05 |
| pTH8982 |  | ceh-48 (0.9) | 63 | 1.90 | 1.4e-05 |
| NKX28\_f1 |  | ceh-24 (0.67) ces-1 C34H4.5 T22H9.4 | 80 | 1.68 | 1.4e-05 |
| I$E74A\_01 |  | lin-1 nhr-19 C24A1.2 | 104 | 1.46 | 1.5e-05 |
| pTH5102 |  | aha-1 (0.59) mxl-2 ref-1 mxl-1 lin-22 hlh-26 hlh-30 C27D6.4 | 73 | 1.75 | 1.6e-05 |
| HES1\_f1 |  | lin-22 | 72 | 1.77 | 1.7e-05 |
| pTH10696 |  | Y44A6D.3 dpy-27 | 58 | 1.98 | 1.7e-05 |
| pTH10650 |  | nhr-153 | 90 | 1.57 | 1.8e-05 |
| E2F4\_1 |  | nfi-1 (0.61) F49E12.6 | 58 | 1.98 | 1.8e-05 |
| HEN1\_si |  | hlh-15 hlh-1 | 29 | 3.09 | 2.0e-05 |
| MA0135.1 |  | lim-7 php-3 cfi-1 lin-39 | 61 | 1.92 | 2.0e-05 |
| ss\_tgo\_SANGER\_10\_FBgn0015014 |  | aha-1 (0.59) ahr-1 | 72 | 1.75 | 2.1e-05 |
| pTH1292 |  | ceh-24 (0.67) pzf-1 | 93 | 1.54 | 2.1e-05 |
| CG31670\_SOLEXA\_5\_FBgn0031375 |  | F21A9.2 CELE\_Y38H8A.5 | 103 | 1.46 | 2.2e-05 |
| V$S8\_01 |  | ceh-45 | 75 | 1.72 | 2.2e-05 |
| pTH7032 |  | F52B11.1 | 66 | 1.83 | 2.4e-05 |
| IRX5\_1 |  | irx-1 | 96 | 1.51 | 2.8e-05 |
| pTH10623 |  | scrt-1 | 73 | 1.72 | 3.2e-05 |
| Gmeb1\_1745 |  | attf-1 | 59 | 1.92 | 3.3e-05 |
| pTH2673 |  | fkh-10 (0.57) let-381 lin-31 C34D1.1 | 111 | 1.40 | 3.3e-05 |
| Antp\_SOLEXA\_FBgn0000095 |  | lim-4 (0.78) ceh-10 (0.52) ceh-36 alr-1 ceh-23 lim-7 mls-2 ceh-53 ceh-31 pha-2 ceh-14 ceh-30 ceh-18 lim-6 ceh-12 ceh-1 egl-5 dsc-1 cog-1 ceh-43 and 8 others  [full list] | 115 | 1.37 | 3.4e-05 |
| Plagl1\_0972 |  | Y53H1A.2 (0.58) | 56 | 1.97 | 3.5e-05 |
| Barx2\_3447 |  | ceh-43 | 72 | 1.73 | 3.5e-05 |
| CG8765\_SANGER\_5\_FBgn0036900 |  | H20J04.3 | 103 | 1.45 | 3.5e-05 |
| pTH4325 |  | ceh-18 | 101 | 1.46 | 3.5e-05 |
| pTH9222 |  | mel-28 | 111 | 1.39 | 4.0e-05 |
| pTH2820 |  | ZC328.2 | 66 | 1.80 | 4.3e-05 |
| pTH9381 |  | ceh-18 | 81 | 1.62 | 4.4e-05 |
| pTH9242 |  | mel-28 | 114 | 1.37 | 4.5e-05 |
| pTH5916 |  | efl-2 (0.54) | 70 | 1.74 | 4.6e-05 |
| Barx1\_2877 |  | ceh-43 | 78 | 1.65 | 4.6e-05 |
| MA0139.1 |  | Y5F2A.4 (0.61) F58G1.2 | 64 | 1.82 | 4.7e-05 |
| PAX8\_f1 |  | pax-2 | 66 | 1.79 | 4.9e-05 |
| pTH6508 |  | nhr-36 (0.51) | 94 | 1.51 | 4.9e-05 |
| sqz\_SOLEXA\_5\_FBgn0010768 |  | mel-28 lin-29 fkh-7 | 123 | 1.31 | 5.0e-05 |
| pTH10031 |  | mbr-1 (0.69) | 18 | 4.42 | 5.1e-05 |
| pTH9934 |  | Y53H1A.2 (0.58) | 75 | 1.68 | 5.5e-05 |
| PTF1A\_f1 |  | lin-32 hlh-2 | 94 | 1.50 | 5.6e-05 |
| FOXB1\_2 |  | ceh-53 let-381 hmg-12 hmbx-1 lin-39 lin-31 Y116A8C.22 | 103 | 1.43 | 5.8e-05 |
| FLI1\_f1 |  | lin-1 | 82 | 1.60 | 5.9e-05 |
| pTH10027 |  | M03D4.4 | 50 | 2.06 | 6.2e-05 |
| V$CDPCR1\_01 |  | ceh-48 (0.9) | 70 | 1.73 | 6.3e-05 |
| pTH9297 |  | ceh-18 | 97 | 1.47 | 6.6e-05 |
| pTH10823 |  | B0310.2 | 82 | 1.60 | 6.8e-05 |
| MA0509.1 |  | daf-19 (0.91) | 80 | 1.61 | 6.9e-05 |
| Pou2f3\_3986 |  | ceh-18 | 92 | 1.51 | 7.1e-05 |
| retn\_SANGER\_5\_FBgn0004795 |  | let-381 ceh-20 cfi-1 | 104 | 1.42 | 7.3e-05 |
| V$OCT1\_03 |  | ceh-18 | 72 | 1.70 | 7.3e-05 |
| pTH9335 |  | mel-28 | 94 | 1.49 | 7.8e-05 |
| pTH8556 |  | pax-2 | 64 | 1.79 | 7.8e-05 |
| pTH9884 |  | tbx-39 | 81 | 1.60 | 7.8e-05 |
| Elf5 |  | C24A1.2 | 99 | 1.45 | 7.9e-05 |
| V$TAXCREB\_01 |  | crh-1 attf-1 | 66 | 1.76 | 8.2e-05 |
| Spdef |  | lin-1 | 81 | 1.60 | 8.2e-05 |
| Hr51\_SANGER\_5\_FBgn0034012 |  | sox-4 (0.62) nhr-100 pop-1 | 93 | 1.50 | 8.4e-05 |
| Msx3\_3206 |  | ceh-10 (0.52) alr-1 ceh-1 lin-39 ceh-45 eyg-1 | 75 | 1.66 | 8.4e-05 |
| pTH8863 |  | hmg-12 | 77 | 1.63 | 8.8e-05 |
| FOXK1\_1 |  | lin-31 nhr-213 | 54 | 1.95 | 9.1e-05 |
| pTH9080 |  | mnm-2 | 73 | 1.67 | 9.2e-05 |
| pTH10037 |  | ref-2 T22C8.4 | 63 | 1.80 | 9.6e-05 |
| V$TATA\_01 |  | tbp-1 | 71 | 1.69 | 9.6e-05 |
| SPDEF\_6 |  | nhr-100 lin-1 lin-39 | 108 | 1.39 | 9.8e-05 |
| Mcm1 |  | unc-120 | 58 | 1.87 | 9.9e-05 |
| Irx6\_2623 |  | irx-1 | 82 | 1.58 | 1.1e-04 |
| pTH8745 |  | attf-1 | 55 | 1.92 | 1.1e-04 |
| pTH9256 |  | ceh-18 | 96 | 1.47 | 1.1e-04 |
| pTH9237 |  | mel-28 | 80 | 1.60 | 1.1e-04 |
| Etv6 |  | lin-1 C24A1.2 | 100 | 1.44 | 1.1e-04 |
| V$GATA1\_03 |  | elt-1 | 81 | 1.59 | 1.1e-04 |
| Vax2\_3500 |  | C02F12.10 | 68 | 1.72 | 1.1e-04 |
| V$OCT1\_06 |  | ceh-18 | 111 | 1.36 | 1.2e-04 |
| Phox2a\_3947 |  | alr-1 ceh-53 ceh-18 ceh-12 ceh-1 npax-3 ceh-43 lin-39 ceh-45 | 79 | 1.60 | 1.2e-04 |
| pTH5423 |  | klf-2 | 78 | 1.61 | 1.2e-04 |
| MA0452.2 |  | B0310.2 ZK177.3 | 86 | 1.54 | 1.3e-04 |
| CEBPE\_f1 |  | C48E7.11 R07H5.10 | 104 | 1.41 | 1.4e-04 |
| V$HOX13\_01 |  | lin-39 | 98 | 1.44 | 1.4e-04 |
| V$CDPCR3\_01 |  | ceh-48 (0.9) ceh-24 (0.67) | 50 | 2.00 | 1.4e-04 |
| Tbp\_pr781 |  | tbp-1 | 102 | 1.42 | 1.5e-04 |
| MA0102.3 |  | C48E7.11 | 72 | 1.66 | 1.5e-04 |
| Emx2\_3420 |  | ceh-2 | 63 | 1.77 | 1.5e-04 |
| Elf3 |  | C24A1.2 | 100 | 1.43 | 1.5e-04 |
| pTH9262 |  | lin-54 | 101 | 1.42 | 1.7e-04 |
| Pou3f3\_3235 |  | ceh-6 | 80 | 1.58 | 1.7e-04 |
| Tcf7l2\_3461 |  | ceh-20 pop-1 | 92 | 1.48 | 1.7e-04 |
| Barhl1\_3 |  | ceh-31 ceh-14 ceh-1 ceh-43 lin-39 | 101 | 1.42 | 1.7e-04 |
| TLX1\_f1 |  | ceh-19 | 74 | 1.63 | 1.8e-04 |
| pTH9958 |  | ztf-2 ztf-6 | 86 | 1.53 | 1.8e-04 |
| Irx2\_0900 |  | irx-1 | 75 | 1.62 | 1.8e-04 |
| MA0544.1 |  | lin-22 gei-11 | 77 | 1.60 | 1.8e-04 |
| HXD9\_f1 |  | hbl-1 (0.79) php-3 lin-39 | 73 | 1.64 | 1.8e-04 |
| Dr\_Cell\_FBgn0000492 |  | ceh-24 (0.67) ceh-9 ceh-8 alr-1 lim-7 ceh-31 ceh-30 ceh-1 ceh-43 ceh-19 lin-39 | 75 | 1.62 | 1.9e-04 |
| TFEC\_1 |  | aha-1 (0.59) hlh-30 | 71 | 1.66 | 1.9e-04 |
| V$FREAC7\_01 |  | lin-31 | 79 | 1.58 | 1.9e-04 |
| ZN384\_f1 |  | K11D2.4 lin-29 | 60 | 1.80 | 2.0e-04 |
| Sox1\_2631 |  | sox-4 (0.62) | 115 | 1.33 | 2.0e-04 |
| YY1\_1 |  | lsy-2 (0.68) | 61 | 1.78 | 2.1e-04 |
| pTH10796 |  | F10B5.3 (0.61) hsf-1 Y53C10A.3 | 99 | 1.43 | 2.1e-04 |
| Lhx4\_1719 |  | ceh-24 (0.67) lim-7 lim-6 ceh-16 | 56 | 1.85 | 2.1e-04 |
| pTH6449 |  | ceh-43 | 66 | 1.71 | 2.1e-04 |
| pTH8333 |  | F39B2.1 (0.62) ZC416.1 | 69 | 1.68 | 2.1e-04 |
| TBX2\_f1 |  | ztf-6 tbx-39 | 86 | 1.52 | 2.2e-04 |
| pTH2842 |  | ceh-24 (0.67) dsc-1 | 55 | 1.86 | 2.3e-04 |
| pnr\_SANGER\_5\_FBgn0003117 |  | elt-1 | 86 | 1.51 | 2.7e-04 |
| pTH8996 |  | hlh-8 daf-8 sma-4 | 86 | 1.51 | 2.7e-04 |
| Pou3f1\_3819 |  | ceh-6 | 85 | 1.52 | 2.7e-04 |
| pTH5169 |  | cfi-1 | 112 | 1.34 | 2.8e-04 |
| Elf3\_3876 |  | C24A1.2 | 101 | 1.40 | 2.8e-04 |
| Vax1\_3499 |  | C02F12.10 | 67 | 1.68 | 2.9e-04 |
| Osr2\_1727 |  | odd-2 odd-1 | 79 | 1.56 | 3.2e-04 |
| pTH9137 |  | nhr-65 | 50 | 1.93 | 3.3e-04 |
| nau\_SANGER\_5\_FBgn0002922 |  | hlh-15 hlh-11 hlh-1 hlh-14 | 81 | 1.54 | 3.3e-04 |
| PO3F2\_si |  | dmd-3 ceh-18 | 109 | 1.35 | 3.3e-04 |
| MA0536.1 |  | elt-1 | 58 | 1.79 | 3.5e-04 |
| pTH9082 |  | mab-23 | 105 | 1.37 | 3.5e-04 |
| pTH9132 |  | ceh-24 (0.67) php-3 pal-1 ceh-13 lin-39 T27F2.4 D1005.3 | 92 | 1.46 | 3.6e-04 |
| HepG2\_HSF1\_Stanford |  | Y53C10A.3 | 74 | 1.60 | 3.7e-04 |
| Irx5\_2385 |  | irx-1 | 72 | 1.62 | 3.8e-04 |
| pTH3997 |  | C04F5.9 | 35 | 2.32 | 3.9e-04 |
| RFX1\_f1 |  | daf-19 (0.91) | 73 | 1.61 | 4.0e-04 |
| pTH10041 |  | ztf-29 | 83 | 1.52 | 4.0e-04 |
| pTH10788 |  | tbx-33 | 98 | 1.41 | 4.2e-04 |
| pTH10816 |  | dmd-6 | 77 | 1.56 | 4.5e-04 |
| Hoxb4\_2627 |  | lin-39 | 81 | 1.53 | 4.6e-04 |
| Ara\_Cell\_FBgn0015904 |  | daf-16 (0.63) fkh-9 irx-1 | 75 | 1.58 | 4.7e-04 |
| pTH1014 |  | atf-5 | 68 | 1.65 | 4.7e-04 |
| pTH9044 |  | mbr-1 (0.69) F26F4.8 nhr-177 bed-3 | 72 | 1.61 | 4.9e-04 |
| pTH6436 |  | ceh-53 | 63 | 1.70 | 4.9e-04 |
| MA0095.2 |  | lsy-2 (0.68) | 96 | 1.42 | 5.0e-04 |
| HeLa-S3\_ZNF274\_UCD |  | C28G1.4 | 64 | 1.69 | 5.0e-04 |
| Meis1\_2335 |  | ceh-32 (0.54) ceh-20 ces-1 F55C5.11 | 81 | 1.52 | 5.1e-04 |
| pTH10654 |  | ceh-90 lsy-27 | 82 | 1.52 | 5.1e-04 |
| pTH6486 |  | nhr-145 (0.52) | 96 | 1.42 | 5.3e-04 |
| pTH10718 |  | egl-43 | 84 | 1.50 | 5.6e-04 |
| pTH2353 |  | D1081.8 B0310.2 lin-48 | 23 | 2.98 | 5.8e-04 |
| pTH9247 |  | dmd-3 C34D1.1 | 104 | 1.36 | 5.8e-04 |
| pTH9135 |  | pop-1 | 64 | 1.68 | 5.9e-04 |
| pTH9164 |  | ceh-26 | 53 | 1.83 | 6.0e-04 |
| rn\_SOLEXA\_5\_FBgn0259172 |  | lin-29 | 105 | 1.36 | 6.1e-04 |
| SRP001585\_Tbx2 |  | lin-1 slr-2 nhr-273 tbx-39 nhr-28 | 68 | 1.63 | 6.5e-04 |
| V$IK2\_01 |  | F26F4.8 | 79 | 1.53 | 6.7e-04 |
| Sox17\_2837 |  | sox-4 (0.62) | 104 | 1.36 | 6.8e-04 |
| Sox11\_2266 |  | sox-4 (0.62) pop-1 | 103 | 1.37 | 7.1e-04 |
| Fli1 |  | lin-1 C24A1.2 | 80 | 1.52 | 7.1e-04 |
| V$CART1\_01 |  | alr-1 ceh-18 ceh-12 pal-1 ceh-43 lin-39 | 87 | 1.46 | 7.4e-04 |
| Hoxc13\_3127 |  | ceh-24 (0.67) pal-1 | 33 | 2.32 | 7.5e-04 |
| Prop1\_3949 |  | ceh-53 ceh-16 | 49 | 1.88 | 7.5e-04 |
| MA0467.1 |  | ceh-45 tbx-39 | 80 | 1.52 | 7.5e-04 |
| Hoxc4\_3491 |  | lin-39 | 80 | 1.51 | 7.7e-04 |
| pTH5257 |  | C48E7.11 | 82 | 1.50 | 7.9e-04 |
| pTH5119 |  | cfi-1 | 59 | 1.72 | 8.0e-04 |
| pTH5891 |  | nhr-49 | 39 | 2.10 | 8.0e-04 |
| MA0495.1 |  | crh-1 jun-1 fos-1 sknr-1 F45H11.6 | 96 | 1.40 | 8.0e-04 |
| Tcf7\_0950 |  | pop-1 | 67 | 1.63 | 8.1e-04 |
| HLH10 |  | hlh-10 unc-120 | 58 | 1.74 | 8.1e-04 |
| MA0072.1 |  | nhr-118 nhr-213 | 72 | 1.58 | 8.2e-04 |
| pTH10772 |  | ceh-52 | 73 | 1.57 | 8.8e-04 |
| pTH8318 |  | attf-1 | 54 | 1.79 | 9.0e-04 |
| pTH6445 |  | ceh-5 | 76 | 1.54 | 9.4e-04 |
| HLH4C\_da\_SANGER\_5\_3\_FBgn0011277 |  | hlh-15 hlh-8 hlh-1 ces-1 hlh-2 | 39 | 2.09 | 9.5e-04 |
| pTH9300 |  | dmd-3 C34D1.1 | 90 | 1.43 | 9.5e-04 |
| Hoxd9\_2 |  | ceh-24 (0.67) php-3 pal-1 lin-39 | 106 | 1.34 | 9.5e-04 |
| pTH7875 |  | mel-28 | 124 | 1.24 | 1.0e-03 |
| V$HLF\_01 |  | ces-2 atf-2 C48E7.11 F23F12.9 Y51H4A.4 | 71 | 1.58 | 1.1e-03 |
| MA0249.1 |  | hlh-15 hlh-8 ngn-1 hlh-32 lin-31 | 55 | 1.76 | 1.1e-03 |
| pTH10013 |  | nhr-168 (-0.54) | 91 | 1.42 | 1.1e-03 |
| MEIS1\_f2 |  | ceh-32 (0.54) lin-32 lin-39 | 94 | 1.41 | 1.1e-03 |
| pTH2283 |  | odd-2 | 94 | 1.41 | 1.1e-03 |
| Dlx2\_2273 |  | ceh-43 | 68 | 1.60 | 1.1e-03 |
| Hoxd1\_3448 |  | ceh-12 | 58 | 1.71 | 1.2e-03 |
| pTH2280 |  | mnm-2 | 109 | 1.32 | 1.2e-03 |
| Mw151 |  | ztf-6 gei-11 C34D1.1 | 111 | 1.31 | 1.2e-03 |
| MA0547.1 |  | skn-1 (0.67) ceh-2 | 112 | 1.30 | 1.2e-03 |
| V$GATA1\_01 |  | elt-1 | 65 | 1.63 | 1.2e-03 |
| pTH1001 |  | dnj-17 | 72 | 1.56 | 1.2e-03 |
| I$DFD\_01 |  | lin-39 | 69 | 1.59 | 1.2e-03 |
| Hoxa4\_3426 |  | lin-39 | 78 | 1.51 | 1.2e-03 |
| Poxm\_SOLEXA\_5\_FBgn0003129 |  | pax-2 | 69 | 1.59 | 1.3e-03 |
| Etv3 |  | lin-1 | 78 | 1.51 | 1.3e-03 |
| pTH9215 |  | C34D1.1 | 93 | 1.41 | 1.3e-03 |
| pTH3041 |  | atf-2 | 67 | 1.60 | 1.5e-03 |
| MA0058.2 |  | mxl-1 irx-1 | 76 | 1.52 | 1.5e-03 |
| pTH6569 |  | ceh-43 | 72 | 1.55 | 1.5e-03 |
| Evx1\_3952 |  | ceh-53 | 61 | 1.66 | 1.5e-03 |
| pTH9254 |  | mel-28 | 116 | 1.28 | 1.5e-03 |
| pTH6497 |  | lin-31 | 93 | 1.40 | 1.5e-03 |
| pTH5118 |  | cfi-1 | 65 | 1.62 | 1.5e-03 |
| pTH5919 |  | irx-1 | 61 | 1.66 | 1.6e-03 |
| Nr2f6\_1 |  | nhr-2 | 70 | 1.57 | 1.6e-03 |
| V$CETS1P54\_02 |  | C52B9.2 | 81 | 1.48 | 1.6e-03 |
| V$FAC1\_01 |  | gei-8 (0.56) | 51 | 1.79 | 1.6e-03 |
| Bsx\_3483 |  | ceh-31 | 62 | 1.64 | 1.6e-03 |
| EN1\_2 |  | ceh-2 ceh-16 | 72 | 1.54 | 1.7e-03 |
| pTH10811 |  | nhr-216 nhr-84 | 89 | 1.42 | 1.7e-03 |
| Elf4 |  | C24A1.2 | 90 | 1.41 | 1.7e-03 |
| SOX10\_2 |  | egl-13 (0.81) sox-4 (0.62) | 100 | 1.35 | 1.8e-03 |
| pTH9043 |  | sem-2 | 93 | 1.39 | 1.8e-03 |
| Eip74EF\_FlyReg\_FBgn0000567 |  | K02D7.2 unc-120 C24A1.2 | 95 | 1.38 | 1.8e-03 |
| pTH8985 |  | athp-1 | 89 | 1.42 | 1.8e-03 |
| Lbx2\_3869 |  | mls-2 | 45 | 1.88 | 1.8e-03 |
| pTH1294 |  | mel-28 | 126 | 1.22 | 1.9e-03 |
| pTH3819 |  | ceh-18 | 80 | 1.48 | 1.9e-03 |
| Lhx1\_2240 |  | lim-7 | 30 | 2.31 | 1.9e-03 |
| pTH5812 |  | ceh-14 | 45 | 1.88 | 1.9e-03 |
| Pou3f4\_3773 |  | ceh-6 | 86 | 1.43 | 2.0e-03 |
| V$GATA3\_01 |  | elt-1 | 63 | 1.62 | 2.0e-03 |
| pTH5877 |  | nhr-100 elt-1 | 90 | 1.41 | 2.1e-03 |
| pTH10808 |  | ztf-19 | 87 | 1.42 | 2.2e-03 |
| Pbx1\_3203 |  | ceh-20 | 66 | 1.58 | 2.3e-03 |
| Atf6\_SANGER\_5\_FBgn0033010 |  | atf-6 (0.76) atf-7 C27D6.4 | 83 | 1.45 | 2.3e-03 |
| Nkx1-1\_3856 |  | ceh-30 | 68 | 1.56 | 2.3e-03 |
| MA0204.1 |  | ceh-32 (0.54) dmd-4 | 69 | 1.55 | 2.4e-03 |
| pTH8998 |  | daf-19 (0.91) mab-3 | 87 | 1.42 | 2.5e-03 |
| MA0016.1 |  | nhr-69 nhr-19 nhr-10 nhr-2 nhr-213 | 60 | 1.64 | 2.6e-03 |
| Hoxa3\_2783 |  | lin-39 | 62 | 1.61 | 2.8e-03 |
| pTH9365 |  | ceh-6 ceh-18 lin-39 | 74 | 1.50 | 2.8e-03 |
| pTH6641 |  | lin-31 | 103 | 1.32 | 2.9e-03 |
| pTH6556 |  | lim-6 odd-1 | 70 | 1.53 | 2.9e-03 |
| pTH9384 |  | cfi-1 | 91 | 1.39 | 2.9e-03 |
| Dlx1\_1741 |  | ceh-43 | 67 | 1.56 | 3.0e-03 |
| pTH9073 |  | end-3 elt-1 | 75 | 1.49 | 3.0e-03 |
| Mw144 |  | elt-1 | 49 | 1.77 | 3.1e-03 |
| MCR\_f1 |  | nhr-255 | 74 | 1.50 | 3.1e-03 |
| NR4A2\_si |  | nhr-6 nhr-71 nhr-213 | 84 | 1.42 | 3.4e-03 |
| MA0260.1 |  | che-1 ces-1 ztf-28 | 81 | 1.44 | 3.4e-03 |
| pTH6636 |  | egl-5 | 79 | 1.46 | 3.5e-03 |
| pTH9261 |  | dmd-3 lin-48 pax-3 | 88 | 1.40 | 3.6e-03 |
| V$EN1\_01 |  | ceh-16 | 63 | 1.58 | 3.7e-03 |
| pTH6003 |  | nhr-134 | 76 | 1.47 | 3.9e-03 |
| V$GR\_Q6 |  | nhr-255 | 85 | 1.41 | 4.0e-03 |
| V$MYB\_Q6 |  | D1081.8 | 70 | 1.52 | 4.0e-03 |
| MA0117.1 |  | pax-2 F45H11.6 | 61 | 1.60 | 4.1e-03 |
| pTH9220 |  | mbr-1 (0.69) | 52 | 1.70 | 4.2e-03 |
| pTH6478 |  | lim-7 | 61 | 1.60 | 4.3e-03 |
| pTH9245 |  | ceh-18 | 88 | 1.39 | 4.3e-03 |
| pTH5250 |  | C48E7.11 | 58 | 1.63 | 4.3e-03 |
| pTH5887 |  | lin-39 | 59 | 1.61 | 4.4e-03 |
| V$YY1\_01 |  | lsy-2 (0.68) | 60 | 1.60 | 4.7e-03 |
| Mafk\_3106 |  | F45H11.6 | 98 | 1.33 | 5.0e-03 |
| Hoxb3\_1720 |  | lin-39 | 63 | 1.56 | 5.1e-03 |
| pTH8983 |  | tag-347 | 76 | 1.46 | 5.3e-03 |
| pTH9925 |  | ztf-11 | 72 | 1.49 | 5.3e-03 |
| Hoxa5\_3415 |  | lin-39 | 62 | 1.57 | 5.4e-03 |
| pTH9709 |  | die-1 | 120 | 1.23 | 5.7e-03 |
| ARI3A\_do |  | gei-3 (0.54) cfi-1 | 149 | 1.07 | 5.8e-03 |
| Hoxd13\_2356 |  | pal-1 | 33 | 2.04 | 6.0e-03 |
| Nkx1-2\_3214 |  | ceh-30 | 67 | 1.52 | 6.5e-03 |
| V$FOXJ2\_02 |  | lin-31 | 53 | 1.66 | 6.5e-03 |
| pTH6071 |  | C46E10.8 C33G8.2 | 74 | 1.46 | 6.5e-03 |
| Tcf3\_3787 |  | pop-1 | 94 | 1.34 | 6.6e-03 |
| pTH9076 |  | C01G12.1 | 21 | 2.60 | 6.7e-03 |
| pTH10630 |  | lsy-27 | 67 | 1.51 | 6.9e-03 |
| BARHL2\_4 |  | ceh-31 | 63 | 1.55 | 6.9e-03 |
| pTH10807 |  | F13H6.1 | 61 | 1.56 | 7.1e-03 |
| Six4\_2860 |  | ceh-32 (0.54) | 54 | 1.63 | 7.3e-03 |
| CG7386\_F10-12\_SANGER\_5\_FBgn0035691 |  | F56D1.1 (0.74) gei-3 (0.54) | 75 | 1.45 | 7.5e-03 |
| pTH6447 |  | ceh-19 | 73 | 1.46 | 7.8e-03 |
| HSFY2\_1 |  | hsf-1 mab-3 | 80 | 1.41 | 8.1e-03 |
| MA0173.1 |  | D1081.8 irx-1 | 54 | 1.63 | 8.2e-03 |
| ECC-1\_ERALPHA\_HudsonAlpha |  | nhr-71 | 60 | 1.56 | 8.3e-03 |
| pTH10030 |  | xbp-1 (0.52) | 66 | 1.51 | 8.4e-03 |
| pTH9198 |  | dmd-3 | 88 | 1.36 | 8.4e-03 |
| pTH8991 |  | cey-3 | 49 | 1.68 | 8.7e-03 |
| GRHL1\_1 |  | grh-1 (-0.65) | 24 | 2.35 | 8.9e-03 |
| pTH6327 |  | dsc-1 | 65 | 1.51 | 8.9e-03 |
| Pknox2\_3077 |  | ceh-32 (0.54) | 69 | 1.48 | 9.0e-03 |
| pTH3477 |  | daf-16 (0.63) | 89 | 1.36 | 9.1e-03 |
| pTH9216 |  | ceh-18 | 61 | 1.55 | 9.1e-03 |
| V$GATA6\_01 |  | elt-1 | 71 | 1.47 | 9.2e-03 |
| Hoxa10\_2318 |  | ceh-24 (0.67) | 140 | 1.13 | 9.8e-03 |
| pTH3084 |  | C01B12.2 attf-1 | 52 | 1.64 | 9.9e-03 |
| pTH8649 |  | mbr-1 (0.69) | 56 | 1.59 | 9.9e-03 |
| pTH5078 |  | ces-2 | 44 | 1.75 | 9.9e-03 |
| pTH9026 |  | attf-1 | 48 | 1.68 | 1.0e-02 |
| I$MTTFA\_01 |  | hmg-5 | 51 | 1.64 | 1.1e-02 |
| pTH9149 |  | ztf-30 | 32 | 1.99 | 1.1e-02 |
| Oli\_da\_SANGER\_5\_2\_FBgn0032651 |  | hlh-32 | 71 | 1.45 | 1.1e-02 |
| pTH5781 |  | ceh-32 (0.54) | 75 | 1.43 | 1.2e-02 |
| ELK3\_f1 |  | lin-1 | 75 | 1.42 | 1.3e-02 |
| pTH9260 |  | mel-28 | 111 | 1.24 | 1.3e-02 |
| Pou2f1\_3081 |  | ceh-18 | 75 | 1.42 | 1.3e-02 |
| V$CEBP\_01 |  | C48E7.11 | 77 | 1.41 | 1.4e-02 |
| V$BRN2\_01 |  | ceh-18 | 99 | 1.29 | 1.4e-02 |
| Cdx2\_4272 |  | ceh-13 | 53 | 1.60 | 1.4e-02 |
| pTH8216 |  | Y116A8C.22 | 62 | 1.51 | 1.5e-02 |
| pTH9974 |  | hlh-16 | 43 | 1.72 | 1.5e-02 |
| Hoxa2\_3079 |  | lin-39 | 61 | 1.51 | 1.5e-02 |
| Meox1\_2310 |  | ceh-31 | 64 | 1.48 | 1.6e-02 |
| pTH10798 |  | Y75B8A.6 | 68 | 1.45 | 1.6e-02 |
| Nkx6-1\_2825 |  | cog-1 | 66 | 1.47 | 1.6e-02 |
| pTH5561 |  | nhr-239 | 53 | 1.58 | 1.7e-02 |
| pTH9911 |  | atf-5 | 72 | 1.43 | 1.7e-02 |
| Gsh2\_3990 |  | ceh-31 | 58 | 1.53 | 1.7e-02 |
| V$TAXCREB\_02 |  | crh-1 zip-3 | 59 | 1.52 | 1.7e-02 |
| Hoxa11\_2218 |  | php-3 | 78 | 1.39 | 1.8e-02 |
| Vsx1\_1728 |  | alr-1 | 35 | 1.85 | 1.8e-02 |
| MA0027.1 |  | ceh-16 | 73 | 1.41 | 1.8e-02 |
| Six2\_2307 |  | ceh-32 (0.54) ceh-34 | 55 | 1.55 | 1.9e-02 |
| srp\_SANGER\_5\_FBgn0003507 |  | elt-1 elt-3 | 83 | 1.35 | 2.0e-02 |
| Hoxa7\_3750 |  | lin-39 | 132 | 1.15 | 2.0e-02 |
| Hoxb5\_3122 |  | lin-39 | 58 | 1.52 | 2.1e-02 |
| pTH9969 |  | pag-3 | 78 | 1.38 | 2.2e-02 |
| NR5A2\_f1 |  | nhr-68 | 70 | 1.42 | 2.2e-02 |
| V$AREB6\_02 |  | ztf-6 | 72 | 1.41 | 2.2e-02 |
| pTH6591 |  | lin-31 | 92 | 1.30 | 2.3e-02 |
| Cutl1\_3494 |  | ceh-44 (0.91) | 64 | 1.46 | 2.3e-02 |
| pTH6482 |  | ceh-19 | 57 | 1.52 | 2.3e-02 |
| Hoxd10\_2368 |  | php-3 | 143 | 1.10 | 2.3e-02 |
| Dlx3\_1030 |  | ceh-43 | 62 | 1.48 | 2.3e-02 |
| Hlxb9\_3422 |  | ceh-12 | 58 | 1.51 | 2.4e-02 |
| Irx3\_0920 |  | irx-1 | 73 | 1.40 | 2.5e-02 |
| pTH6562 |  | ceh-5 | 59 | 1.50 | 2.5e-02 |
| V$T3R\_01 |  | nhr-15 | 58 | 1.50 | 2.5e-02 |
| pTH10777 |  | dmd-3 | 107 | 1.24 | 2.5e-02 |
| pTH3751 |  | tbx-39 | 73 | 1.40 | 2.6e-02 |
| pTH5437 |  | ceh-34 | 111 | 1.22 | 2.6e-02 |
| Hmx1\_3423 |  | ceh-9 | 47 | 1.61 | 2.6e-02 |
| pTH6423 |  | pha-2 | 70 | 1.41 | 2.8e-02 |
| ATF1\_si |  | crh-1 | 70 | 1.41 | 2.8e-02 |
| V$CDXA\_01 |  | php-3 ceh-13 | 75 | 1.38 | 2.8e-02 |
| Cdx1\_2245 |  | ceh-13 | 139 | 1.11 | 3.1e-02 |
| pTH10028 |  | nhr-204 (-0.53) | 50 | 1.56 | 3.3e-02 |
| pTH6106 |  | nhr-182 | 73 | 1.38 | 3.4e-02 |
| Tcf2\_0913 |  | hmbx-1 | 56 | 1.50 | 3.5e-02 |
| Tcf1\_2666 |  | hmbx-1 | 11 | 3.29 | 3.8e-02 |
| pTH3046 |  | Y116A8C.22 | 77 | 1.35 | 4.0e-02 |
| Jundm2\_0911 |  | fos-1 | 54 | 1.50 | 4.1e-02 |
| Hoxa6\_1040 |  | lin-39 | 62 | 1.43 | 4.4e-02 |
| TCF4\_2 |  | hlh-2 | 70 | 1.38 | 4.4e-02 |
| MA0146.2 |  | F58G1.2 | 48 | 1.55 | 4.5e-02 |
| pTH2846 |  | lin-31 | 87 | 1.29 | 4.7e-02 |
| pTH9708 |  | ceh-34 | 27 | 1.91 | 4.7e-02 |
| pTH10805 |  | ztf-16 | 44 | 1.59 | 4.8e-02 |
| Spdef\_0905 |  | lin-1 | 64 | 1.41 | 4.9e-02 |
| pTH6268 |  | ceh-2 | 40 | 1.64 | 5.0e-02 |
| pTH5778 |  | egl-5 | 63 | 1.42 | 5.0e-02 |

### Correlated (and anti-correlated) transcription factors

|  |  |
| --- | --- |
| **Transcription factor** | **Correlation** |
| nhr-47 | 0.95 |
| C34F6.9 | 0.94 |
| Y17G7B.22 | 0.93 |
| daf-19 | 0.91 |
| ceh-44 | 0.91 |
| sem-4 | 0.90 |
| ceh-48 | 0.90 |
| spr-4 | 0.88 |
| C33H5.17 | 0.88 |
| dhhc-14 | 0.88 |
| Y37F4.6 | 0.87 |
| ctbp-1 | 0.87 |
| ceh-58 | 0.84 |
| rbr-2 | 0.83 |
| dpff-1 | 0.83 |
| syd-9 | 0.82 |
| daf-3 | 0.82 |
| gei-17 | 0.82 |
| sta-1 | 0.82 |
| ceh-38 | 0.82 |
| egl-13 | 0.81 |
| F27D4.6 | 0.81 |
| miz-1 | 0.81 |
| nhr-48 | 0.80 |
| ZK546.5 | 0.80 |
| nhr-60 | -0.42 |
| nhr-5 | -0.42 |
| atf-8 | -0.42 |
| hlh-12 | -0.43 |
| T26A5.8 | -0.43 |
| nhr-141 | -0.43 |
| nhr-156 | -0.44 |
| ceh-7 | -0.44 |
| Y48A6C.1 | -0.45 |
| hlh-29 | -0.45 |
| nhr-112 | -0.46 |
| ztf-27 | -0.46 |
| nhr-92 | -0.46 |
| nhr-207 | -0.47 |
| nhr-70 | -0.47 |
| nhr-146 | -0.47 |
| zip-6 | -0.52 |
| nhr-204 | -0.53 |
| Y56A3A.18 | -0.53 |
| nhr-168 | -0.54 |
| ceh-82 | -0.56 |
| nhr-104 | -0.58 |
| nhr-90 | -0.60 |
| grh-1 | -0.65 |
| nhr-122 | -0.69 |

### ChIP peaks enriched

|  |  |  |  |  |
| --- | --- | --- | --- | --- |
| **Gene** | **Experiment** | **Number of upstream peaks** | **Enrichment** | **FDR corrected p** |
| ces-1 | CES-1\_Embryos | 103 | 2.90 | 1.2e-28 |
| ces-1 | CES-1\_Fed-L1-stage-larvae | 64 | 4.67 | 5.8e-25 |
| C34F6.9 | C34F6.9\_Larvae-L2-stage | 96 | 2.77 | 6.2e-24 |
| F45C12.2 | F45C12.2\_Fed-L1-stage-larvae | 87 | 3.06 | 1.4e-23 |
| zag-1 | ZAG-1\_Larvae-L2-stage | 75 | 3.46 | 2.8e-22 |
| ham-1 | HAM-1\_Larvae-L4-stage | 93 | 2.59 | 1.3e-20 |
| nfya-1 | NFYA-1\_Late-Embryos | 85 | 2.79 | 4.3e-20 |
| eor-1 | EOR-1\_Larvae-L3-stage | 89 | 2.64 | 8.8e-20 |
| ceh-26 | CEH-26\_Late-Embryonic-stage | 65 | 3.65 | 1.2e-19 |
| lin-35 | LIN-35\_Fed-L1-stage-larvae | 79 | 2.70 | 3.4e-17 |
| alr-1 | ALR-1\_Larvae-L2-stage | 69 | 2.85 | 2.1e-15 |
| nfya-1 | NFYA-1\_Larvae-L3-stage | 71 | 2.75 | 3.4e-15 |
| gei-11 | GEI-11\_Larvae-L3-stage | 68 | 2.85 | 3.8e-15 |
| sem-4 | SEM-4\_Larvae-L2-stage | 82 | 2.40 | 5.5e-15 |
| gei-11 | GEI-11\_Fed-L1-stage-larvae | 77 | 2.42 | 7.0e-14 |
| efl-1 | EFL-1\_Fed-L1-stage-larvae | 73 | 2.53 | 7.3e-14 |
| sea-2 | SEA-2\_Larvae-L3-stage | 33 | 5.66 | 7.9e-14 |
| dpl-1 | DPL-1\_Larvae-L4-stage | 93 | 2.05 | 1.5e-13 |
| sax-3 | SAX-3\_Larvae-L2-stage | 58 | 3.04 | 1.5e-13 |
| lsy-2 | LSY-2\_Fed-L1-stage-larvae | 64 | 2.75 | 3.1e-13 |
| egl-5 | EGL-5\_Larvae-L3-stage | 65 | 2.68 | 5.7e-13 |
| lsy-2 | LSY-2\_Larvae-L1-stage | 76 | 2.35 | 6.9e-13 |
| efl-1 | EFL-1\_Larvae-L1-stage | 77 | 2.31 | 9.1e-13 |
| ces-1 | CES-1\_Larvae-L3-stage | 46 | 3.56 | 1.6e-12 |
| W03F9.2 | W03F9.2\_L4-Young-Adult-stage-larvae | 100 | 1.86 | 2.7e-12 |
| zag-1 | ZAG-1\_Larvae-L3-stage | 42 | 3.78 | 4.2e-12 |
| pes-1 | PES-1\_Larvae-L4-stage | 77 | 2.24 | 4.6e-12 |
| fos-1 | FOS-1\_Fed-L1-stage-larvae | 73 | 2.33 | 4.7e-12 |
| zag-1 | ZAG-1\_Larvae-L4-stage | 42 | 3.76 | 5.0e-12 |
| gei-11 | GEI-11\_Larvae-L2-stage | 61 | 2.65 | 9.2e-12 |
| zag-1 | ZAG-1\_Fed-L1-stage-larvae | 37 | 4.11 | 1.6e-11 |
| ham-1 | HAM-1\_Fed-L1-stage-larvae | 73 | 2.27 | 2.0e-11 |
| F45C12.2 | F45C12.2\_Larvae-L2-stage | 33 | 4.55 | 2.8e-11 |
| ztf-7 | ZTF-7\_Larvae-L4-stage | 54 | 2.84 | 3.3e-11 |
| skn-1 | SKN-1\_Larvae-L3-stage | 42 | 3.51 | 4.5e-11 |
| aly-2 | ALY-2\_Fed-L1-stage-larvae | 61 | 2.55 | 4.6e-11 |
| lsy-2 | LSY-2\_Embryos | 59 | 2.58 | 8.9e-11 |
| sax-3 | SAX-3\_Larvae-L4-stage | 70 | 2.26 | 1.0e-10 |
| ceh-38 | CEH-38\_Larvae-L4-stage | 48 | 3.01 | 1.5e-10 |
| mab-5 | MAB-5\_Larvae-L2-stage | 40 | 3.52 | 1.7e-10 |
| R02D3.7 | R02D3.7\_Larvae-L3-stage | 76 | 2.11 | 1.7e-10 |
| lsy-2 | LSY-2\_Larvae-L4-stage | 41 | 3.41 | 2.1e-10 |
| ces-1 | CES-1\_Larvae-L4-stage | 42 | 3.25 | 4.7e-10 |
| dpl-1 | DPL-1\_Fed-L1-stage-larvae | 67 | 2.24 | 7.3e-10 |
| lin-13 | LIN-13\_Larvae-L2-stage | 55 | 2.52 | 1.8e-09 |
| lin-15 | LIN-15B\_Fed-L1-stage-larvae | 47 | 2.81 | 2.5e-09 |
| F45C12.2 | F45C12.2\_Larvae-L3-stage | 39 | 3.26 | 2.9e-09 |
| hpl-2 | HPL-2\_Fed-L1-stage-larvae | 77 | 1.98 | 2.9e-09 |
| nhr-237 | NHR-237\_Embryos | 39 | 3.21 | 4.5e-09 |
| ceh-39 | CEH-39\_Embryos | 40 | 3.13 | 5.2e-09 |
| nhr-129 | NHR-129\_Larvae-L2-stage | 73 | 2.01 | 6.4e-09 |
| hlh-30 | HLH-30\_Late-Embryos | 41 | 2.97 | 1.3e-08 |
| ceh-38 | CEH-38\_Larvae-L3-stage | 53 | 2.41 | 2.2e-08 |
| jun-1 | JUN-1\_Larvae-L1-stage | 47 | 2.62 | 2.4e-08 |
| nhr-6 | NHR-6\_Larvae-L2-stage | 55 | 2.31 | 4.2e-08 |
| unc-39 | UNC-39\_Larvae-L2-stage | 20 | 5.62 | 4.8e-08 |
| sax-3 | SAX-3\_Fed-L1-stage-larvae | 21 | 4.99 | 1.3e-07 |
| nhr-77 | NHR-77\_Fed-L1-stage-larvae | 55 | 2.22 | 1.7e-07 |
| lin-13 | LIN-13\_Larvae-L4-stage | 43 | 2.60 | 2.1e-07 |
| efl-1 | EFL-1\_Young-adult | 70 | 1.90 | 2.9e-07 |
| nhr-77 | NHR-77\_Larvae-L3-stage | 53 | 2.23 | 3.3e-07 |
| sax-3 | SAX-3\_Larvae-L3-stage | 25 | 3.95 | 3.5e-07 |
| pax-1 | PAX-1\_Embryos | 22 | 4.37 | 5.5e-07 |
| hlh-30 | HLH-30\_Larvae-L4-stage | 37 | 2.77 | 6.8e-07 |
| elt-1 | ELT-1\_Larvae-L3-stage | 36 | 2.81 | 7.9e-07 |
| R02D3.7 | R02D3.7\_Larvae-L2-stage | 20 | 4.58 | 1.2e-06 |
| pha-4 | PHA-4\_Larvae-L2-stage | 70 | 1.82 | 1.6e-06 |
| mef-2 | MEF-2\_Fed-L1-stage-larvae | 21 | 4.18 | 2.4e-06 |
| unc-62 | UNC-62\_Larvae-L2-stage | 21 | 4.16 | 2.6e-06 |
| nfya-1 | NFYA-1\_Young-adult | 27 | 3.32 | 2.7e-06 |
| dve-1 | DVE-1\_Late-Embryos | 45 | 2.25 | 5.4e-06 |
| unc-62 | UNC-62\_Day-Four-Young-Adult | 37 | 2.54 | 5.7e-06 |
| unc-62 | UNC-62\_Young-adult-Day-4 | 37 | 2.54 | 5.7e-06 |
| gei-11 | GEI-11\_Young-adult | 31 | 2.87 | 5.9e-06 |
| ztf-11 | ZTF-11\_Embryos | 13 | 6.62 | 7.0e-06 |
| lsy-2 | LSY-2\_Larvae-L2-stage | 34 | 2.64 | 8.4e-06 |
| nhr-237 | NHR-237\_Larvae-L1-stage | 22 | 3.68 | 9.3e-06 |
| F16B12.6 | F16B12.6\_Fed-L1-stage-larvae | 34 | 2.62 | 1.0e-05 |
| fkh-2 | FKH-2\_Larvae-L3-stage | 19 | 4.17 | 1.1e-05 |
| unc-62 | UNC-62\_Larvae-L3-stage | 37 | 2.47 | 1.1e-05 |
| nhr-77 | NHR-77\_Larvae-L4-stage | 72 | 1.70 | 1.4e-05 |
| C01B12.2 | C01B12.2\_Larvae-L2-stage | 75 | 1.66 | 1.5e-05 |
| gei-11 | GEI-11\_Embryos | 26 | 3.09 | 1.8e-05 |
| jun-1 | JUN-1\_Larvae-L4-stage | 44 | 2.17 | 2.0e-05 |
| dpl-1 | DPL-1\_Young-adult | 55 | 1.92 | 2.1e-05 |
| nhr-6 | NHR-6\_Larvae-L4-stage | 20 | 3.74 | 2.6e-05 |
| pha-4 | PHA-4\_Larvae-L4-stage | 34 | 2.47 | 3.8e-05 |
| C16A3.4 | C16A3.4\_Fed-L1-stage-larvae | 39 | 2.24 | 5.4e-05 |
| fos-1 | FOS-1\_Larvae-L2-stage | 52 | 1.90 | 7.0e-05 |
| aha-1 | AHA-1\_Larvae-L4-stage | 21 | 3.29 | 9.7e-05 |
| unc-62 | UNC-62\_Fed-L1-stage-larvae | 19 | 3.55 | 1.0e-04 |
| nhr-23 | NHR-23\_Larvae-L3-stage | 53 | 1.85 | 1.3e-04 |
| daf-12 | DAF-12\_Larvae-L3-stage | 11 | 6.03 | 1.4e-04 |
| F23B12.7 | F23B12.7\_Young-adult | 38 | 2.16 | 1.7e-04 |
| fos-1 | FOS-1\_Larvae-L3-stage | 48 | 1.91 | 1.8e-04 |
| elt-3 | ELT-3\_Embryos | 34 | 2.28 | 2.0e-04 |
| skn-1 | SKN-1\_Larvae-L4-stage | 9 | 7.04 | 3.2e-04 |
| nhr-25 | NHR-25\_Larvae-L2-stage | 42 | 1.98 | 3.6e-04 |
| jun-1 | JUN-1\_Larvae-L3-stage | 32 | 2.26 | 4.5e-04 |
| pha-4 | PHA-4\_Young-adult | 28 | 2.42 | 5.5e-04 |
| ztf-4 | ZTF-4\_Larvae-L3-stage | 16 | 3.51 | 7.1e-04 |
| dve-1 | DVE-1\_Larvae-L4-stage | 35 | 2.09 | 8.5e-04 |
| nhr-77 | NHR-77\_Larvae-L2-stage | 16 | 3.23 | 1.8e-03 |
| nhr-11 | NHR-11\_Larvae-L2-stage | 26 | 2.34 | 1.9e-03 |
| nhr-2 | NHR-2\_Embryos | 26 | 2.29 | 2.5e-03 |
| nhr-28 | NHR-28\_Larvae-L4-stage | 66 | 1.51 | 2.9e-03 |
| fos-1 | FOS-1\_Larvae-L4-stage | 22 | 2.47 | 3.3e-03 |
| aly-2 | ALY-2\_Larvae-L2-stage | 16 | 3.03 | 3.6e-03 |
| ceh-16 | CEH-16\_Larvae-L2-stage | 21 | 2.50 | 4.1e-03 |
| aly-2 | ALY-2\_Larvae-L3-stage | 14 | 2.97 | 1.1e-02 |
| ztf-4 | ZTF-4\_Larvae-L2-stage | 19 | 2.35 | 1.7e-02 |
| peb-1 | PEB-1\_Larvae-L2-stage | 16 | 2.51 | 2.3e-02 |
| unc-39 | UNC-39\_Embryos | 11 | 3.20 | 2.4e-02 |
| R02D3.7 | R02D3.7\_Larvae-L4-stage | 26 | 1.94 | 2.7e-02 |
| lin-13 | LIN-13\_Larvae-L1-stage | 17 | 2.36 | 3.0e-02 |
| nhr-23 | NHR-23\_Larvae-L2-stage | 10 | 3.19 | 4.1e-02 |
| ama-1 | AMA-1\_Larvae-L3-stage | 23 | 1.95 | 4.8e-02 |
